# Fluoropolymer Coated DNA Nanoclews for Volumetric Visualization of Oligonucleotides Delivery and Near Infrared Light Activated Anti‐Angiogenic Oncotherapy

**DOI:** 10.1002/advs.202304633

**Published:** 2023-09-28

**Authors:** Peng Zhang, Ranran Guo, Haiting Zhang, Wuli Yang, Ye Tian

**Affiliations:** ^1^ Biomaterials Research Center School of Biomedical Engineering Guangdong Provincial Key Laboratory of Construction and Detection in Tissue Engineering Southern Medical University Guangzhou 510515 China; ^2^ School of Biomedical Engineering Guangzhou Medical University Guangzhou 510182 China; ^3^ State Key Laboratory of Molecular Engineering of Polymers & Department of Macromolecular Science Fudan University Shanghai 200438 China

**Keywords:** angiogenesis suppression, DNA nanostructures, gene therapy, oligonucleotides delivery, volumetric imaging

## Abstract

The potential of microRNA regulation in oncotherapy is limited by the lack of delivery vehicles. Herein, it is shown that fluoropolymer coated DNA nanoclews (FNCs) provide outstanding ability to deliver oligonucleotide through circulation and realize near infrared (NIR) light activated angiogenesis suppression to abrogate tumors. Oligonucleotides are loaded in DNA nanoclews through sequence specific bindings and then a fluorinated zwitterionic polymer is coated onto the surface of nanoclews. Further incorporating quantum dots in the polymer coating endows the vectors with NIR‐IIb (1500–1700 nm) fluorescence and NIR light triggered release ability. The FNC vector can deliver oligonucleotides to cancer cells systemically and realize on‐demand cytosolic release of the cargo with high transfection efficiency. Taking advantage of the NIR‐IIb emission, the whole delivery process of FNCs is visualized volumetrically in vivo with a NIR light sheet microscope. Loaded by FNCs, an oligonucleotide can effectively silence the target miRNA when activated with NIR light, and inhibit angiogenesis inside tumor, leading to complete ablation of cancer. These findings suggest FNCs can be used as an efficient oligonucleotide delivery platform to modulate the expression of endogenous microRNA in gene therapy of cancer.

## Graphical abstract

Here delivering oligonucleotides with fluoropolymer coated DNA nanoclews is presented, which can be visualized noninvasively via 3D near infrared (NIR) fluorescence imaging. When activated by NIR light, cargos are released in the cytoplasm with high transfection efficiency, inhibiting tumor angiogenesis. This strategy offers an oligonucleotide delivery platform for microRNA regulation therapy and a novel paradigm in gene vector design.

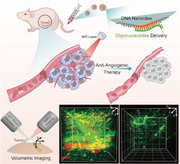

## 1 Introduction

MicroRNAs (miRNAs), a diverse class of short regulatory RNAs, have been proved to play a critical role in the onset and progress of cancer.^[^ ^1^ ^]^ Regulation of aberrantly expressed miRNAs has thus become an effective genetic therapeutic strategy for oncotherapy and attracted much research and clinical attention in the past decade.^[^ ^2^ ^]^ However, its success is largely dependent on the delivery efficiency due to the poor circulation stability and nonspecific biodistribution of the short RNA sequences.^[^ ^3^ ^]^ Various nonviral vectors with low immunogenicity and potential for large scale manufacture have been developed to encapsulate nucleic acids, prolong blood circulation time, target tumor sites specifically and overcome biological barriers.^[^ ^4^ ^]^ These vehicles generally employ cationic polyelectrolytes, including dendrimers, micelles and polymeric nanoparticles, to neutralize the negative charges of nucleic acids and the strong multivalent electrostatic interactions prevent the complex dissociation, which significantly interferes transfection efficiency.^[^ ^5^ ^]^ Therefore, many efforts are devoted in recent years to molecular designs of synthetic vectors that are able to facilitate nucleic acids release by degradation or charge reversal^[^ ^4^, ^6^ ^]^ upon endogenous/exogenous stimuli. Besides, self‐assembled nucleic acid structures, such as hydrogels^[^ ^3^ ^]^ and nanogels,^[^ ^7^ ^]^ have also demonstrated promising applicability in the delivery of oligonucleotides. In these strategies, small nucleic acid sequences are stably linked to the backbone through hydrogen bonds between base pairs, a relatively weak interaction that is facile for dissociation.^[^ ^8^ ^]^ Nevertheless, the nucleic acid structures are still highly negatively charged and thus can only be used in the local delivery of therapeutic sequences.^[^ ^3^, ^7^ ^]^ Hence, a nanoscale delivery platform with long circulation time, superior endocytosis behavior and boosted transfection efficiency is prerequisite to fulfill the therapeutic potential of miRNA regulation.

To address the limitations associated with current oligonucleotide delivery vehicles, we develop fluoropolymer coated DNA nanoclews (FNCs) for the systemic delivery of oligonucleotides. DNA nanoclews, synthesized by rolling circle amplification (RCA),^[^ ^9^ ^]^ with sequence specific binding sites can adsorb oligonucleotides through Watson‐Crick hydrogen bonds. Fluorinated zwitterionic monomers are polymerized in situ and attached to the DNA nanoclews also through hydrogen bonding. The fluoropolymer coating prolongs circulation time^[^ ^10^ ^]^ and facilitates cellular internalization and endosomal escape^[^ ^11^ ^]^ of the DNA nanoclews. After incorporating NIR‐IIb (1500–1700 nm) emitting core‐shell PbS@CdS quantum dots (CSQDs)^[^ ^12^ ^]^ in the polymer coating, the in vivo delivery process is visualized volumetrically at cellular scale by noninvasive optical sectioning using a near infrared light sheet microscope (NIR‐LSM).^[^ ^13^ ^]^ Furthermore, the photothermal effect of quantum dots afford NIR light triggered hydrogen bonding dissociation, leading to detachment of fluoropolymer coating and on‐demand release of cargo oligonucleotides simultaneously. Antisense miR‐21 oligonucleotides (denoted as anti21) are chosen as the therapeutic sequence in the present work and delivered to cancer cells with FNCs, since silencing miR‐21 enables inhibition of vascular endothelial growth factor (VEGF) secretion and suppression of angiogenesis in the tumor.^[^ ^14^ ^]^ After delivered effectively to breast cancer cells through circulation in a humanized orthotropic cancer model, anti21 is readily released in the cytosol upon NIR light illumination. High transfection efficiency achieves successful miRNA regulation and anti‐angiogenesis therapy and results in the ablation of tumor, highlighting great potential of this oligonucleotide delivery platform for improving therapeutic efficacy in gene therapy.

## 2 Results and Discussion

### 2.1 Design, Synthesis, and Characterization

**Figure 1:**
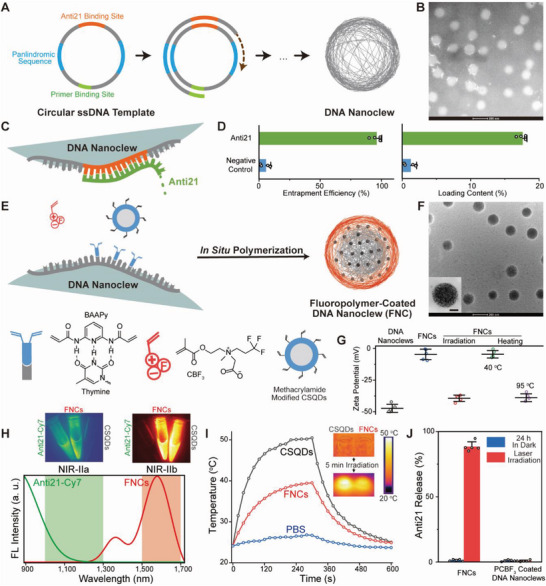
A) Synthesis of DNA nanoclews from circular ssDNA template by RCA. B) TEM image of DNA nanoclews (negatively stained with phosphotungstic acid). C) Anti21 loaded onto DNA nanoclews through sequence specific binding. D) Entrapment efficiencies and loading contents of anti21 and a random ssRNA sequence (negative control) in DNA nanoclews. E) Schematic illustration of fluoropolymer coating to form FNCs: DNA nanoclews are conjugated with BAAPy through triple hydrogen bonds; fluorinated zwitterionic monomer CBF_3_ are polymerized in situ to form the polymer coating; methacrylamide modified CSQDs are also added in the monomer solution so that they can be incorporation in the polymer coating. The diagram is not drawn to scale. F) TEM image of FNCs. The inset image shows the distribution of CSQDs in FNCs (scale bar: 50 nm). G) Zeta potentials of DNA nanoclews and FNCs. After NIR laser irradiation or heating at 95 °C for 5 min, the zwitterionic polymer coating detach from DNA nanoclews, leading to the decrease of zeta potential. Heating at 40 °C cannot peel off the polymer coating. H) NIR‐IIa (up, left, excited with 660 nm laser) and NIR‐IIb (up, right, excited with 808 nm laser) fluorescence images of anti21‐Cy7, FNCs and CSQDs. Fluorescence emission spectra (down) of anti21‐Cy7 (excited with 660 nm laser) and FNCs (excited with 808 nm laser). I) Photothermal response of CSQDs and FNCs at an equivalent concentration (2 mg mL^−1^) with a NIR laser (808 nm, 0.33 W cm^−2^). Irradiation is continued for 300 s, and then the laser is turned off. PBS is used as a negative control. Inset: Thermal images of CSQDs and FNCs before and after NIR laser irradiation. J) Anti21 release profiles of FNCs and PCBF_3_‐coated DNA nanoclews (in the absence of CSQDs) with or without NIR laser irradiation.

The DNA nanoclews are synthesized by RCA with circular single strand DNA (ssDNA) templates, which include palindromic sequences to drive the self‐assembly into nanoclews^[^ ^15^ ^]^ and part sequence of anti21 as binding sites (schemed in **Figure** **1**A, detailed sequence shown in Table S1, Supporting Information). The as‐prepared DNA nanoclews demonstrate a uniform spherical shape in the transmission electron microscope (TEM) with a hydrodynamic diameter of 211 nm (Figure 1B and Figure S1A, Supporting Information). Anti21 is then loaded in the nanoclews through sequence specific binding (Figure 1C). As quantitatively characterized with NIR fluorescence of cyanine dye Cy7 labeled on the oligonucleotides (Figure S2, Supporting Information), the entrapment efficiency of anti21 is above 90%, and loading content achieves 17%, while the entrapment efficiency and loading content of a random sequence is much lower (Figure 1D). Anti21 loading slightly increases the size of DNA nanoclews and has no significant influence on their morphology (Figure S1A,B, Supporting Information). Regulating the reaction time of RCA leads to nanoclews with different size,^[^ ^16^ ^]^ while the entrapment efficiency is independent on the nanoclew size (Figure S1C,D, Supporting Information).

Fluorinated carboxybetaine monomer (CBF_3_) and bis(acrylamide)pyridine (BAAPy) are then copolymerized in situ to form fluoropolymer (PCBF_3_) coatings on the surface of DNA nanoclews (schemed in Figure 1E). BAAPy binds to thymine base in DNA nanoclews through triple hydrogen bonds^[^ ^17^ ^]^ and also acts as cross‐linkers in the polymer coatings. The carboxybetaine zwitterionic nature of PCBF_3_ endows DNA nanoclews anti‐fouling ability, extended blood circulation time and negligible accelerated blood clearance phenomenon after administration in living mammals.^[^ ^18^ ^]^ Compared with conventional zwitterionic polymers, e.g. poly(carboxybetaine methacrylate) (PCBMA), fluorination further improves the cellular uptake of DNA nanoclews and enables endosomal escape.^[^ ^11a^ ^]^ Pre‐mixed with CBF_3_ monomers, methacrylamide modified CSQDs are incorporated in the PCBF_3_ coatings through a polymerization induced self‐assembly process, forming FNCs with a raspberry‐like morphology (shown in Figure 1F). After PCBF_3_ coating, the hydrodynamic diameter increases to 256 nm and the zeta potential of DNA nanoclews increases from −47 to −5 mV, indicating the electrically neutral zwitterionic polymers covering the negatively charged DNAs (Figure 1G).^[^ ^19^ ^]^ PCBF_3_ coating provides DNA nanoclews with improved stability against nuclease and serum, which is suggested by agarose gel electrophoresis (Figure S3, Supporting Information). The zwitterionic PCBF_3_ coatings are also inherently resistant to nonspecific protein adsorption, which is benefit to inhibit the formation of protein corona and clearance by reticuloendothelial systems during blood circulation.^[^ ^18b^ ^]^ Incubated with human serum albumin (HSA) for 1 week, PCBF_3_ coated DNA nanoclews showed no obvious variation in hydrodynamic diameter and zeta potential, while negatively charged DNA nanoclews adsorb proteins immediately when mixed with HSA, indicating excellent anti‐fouling ability of the zwitterionic polymer (Figure S4, Supporting Information).

Since CSQDs are incorporated in the fluoropolymer coatings, the FNCs also emit NIR‐IIb fluorescence,^[^ ^12^ ^]^ whose fluorescence spectrum is shown in Figure 1H. Under an 808‐nm laser excitation, the emission peak of FNCs locates at 1580 nm, falling in the 1500–1700 nm NIR‐IIb region, and thus in vivo distribution of FNCs can be tracked with NIR fluorescence imaging. Although CSQDs exhibit bright NIR‐IIb fluorescence, the absolute quantum yield is only ≈2.0% (Figure S5, Supporting Information),^[^ ^20^ ^]^ indicating the loss of most absorbed light energy. Since vibration is often the preferred route for non‐radiative transition,^[^ ^21^ ^]^ we further characterize the photothermal effect of CSQDs and find out that the quantum dots are an effective photothermal conversion (PTC) agent with a PTC efficiency of 63.7% (Figure 1I). Therefore, CSQDs in FNCs can generate mild heat upon long‐term NIR light illumination, which can be employed to cleave hydrogen bonding between base pairs in DNA nanoclews. After laser illumination at a power density of 330 mW cm^−2^ for 300 s, temperature of FNC dispersion increases to 40 °C from room temperature. The PTC efficiency of FNCs is calculated to be 32.4% and the low PTC efficiency of FNCs when compared to that of CSQDs indicates a new pathway to dissipate the absorbed light energy, which possibly results from hydrogen bond cleavage. Zeta potential decreases back to −39 mV (Figure 1G), indicating detachment of PCBF_3_ coating and exposure of negatively charged DNA nanoclews. The exfoliation of fluoropolymer can also be identified using gel permeation chromatography and ^19^F‐NMR spectra (Figure S6, supporting information). Since the heat sources, CSQDs, are closely linked to the nanoclew core, locally generated heat upon laser illumination can readily destroy hydrogen bonds between BAAPy and thymine,^[^ ^8^, ^22^ ^]^ leading to deconstruction of PCBF_3_ coating from the nanoclews. In a control experiment, the structure of FNCs maintains stable until the dispersion is directly heated up to 95 °C, which is a commonly used temperature in molecular biology to break down base pairing. Photothermal triggered hydrogen bond cleavage also leads to the release of cargo oligonucleotides from the nanoclews, which is shown in Figure 1J. Release of anti21 is below 2% during 24 h, demonstrating the stability of loading oligonucleotides with Watson‐Crick hydrogen bonds. Whereas, above 90% of loaded anti21 is released 5 min after laser illumination, achieving on‐demand release of cargo. It should be noted that compared with NIR laser illumination, the weak excitation light (60 mW cm^−2^) and short exposure time (20 to 100 milliseconds) for NIR fluorescence imaging is far from generating enough heat to trigger oligonucleotide release (Figure S7, Supporting Information). These results show that FNCs possess excellent NIR light triggered oligonucleotide release capability and can be employed as an effective delivery platform in gene therapy.

### 2.2 Cellular Internalization and Cytosolic Release

To exclude any cytotoxicity associated with anti21, DNA nanoclews and PCBF_3_ coatings, cell viability of human breast adenocarcinoma (MCF‐7) cell line is examined by cell counting kit 8 (CCK8) assay. As shown in Figure S8 (Supporting Information), cells maintain high viability after treated with various concentrations of anti21, DNA nanoclews and FNCs, respectively. The phototoxicity of FNCs is also studied: under high concentrations, viability inhibition is observed after NIR light illumination, resulting from the generated mild hyperthermia. At a concentration of 0.5 mg mL^−1^, the cells keep ≈100% viability, which was chosen for subsequent in vitro experiments.

**Figure 2:**
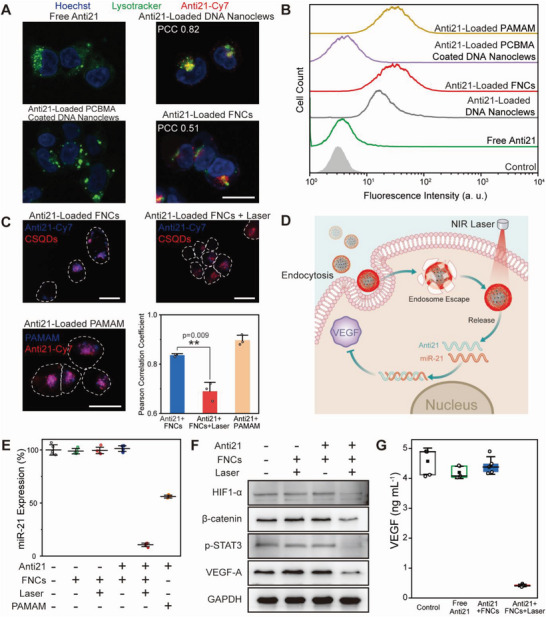
A) Confocal microscopic images of MCF‐7 cells incubated with free Anti21 and Anti21‐loaded vectors. White bar indicates 20 µm in the image. Cells are costained with Lysotracker to observe the endosomal escape of cargo oligonucleotides. PCCs are used to quantify the fluorescence signal overlap of Lysotracker and Anti21. B) Representative histograms of cells incubated with free Anti21 and Anti21‐loaded vectors. C) NIR‐II microscopic images of MCF‐7 cells incubated with Anti21‐loaded FNCs before and after laser illumination. CLSM image of Anti21 loaded with PAMAM is used as a negative control to show cytosolic release of oligonucleotides from vectors. Scale bars indicate 20 µm in all the three images. D) Schematic diagram of cell internalization, endosomal escape and cytosolic release upon NIR laser irradiation of FNCs. The released oligonucleotides can silence miR‐21 and downregulate the secretion of VEGF. E) Quantitative RT‐PCR assay for miR‐21 in MCF‐7 cells 24 h after treatments. F) Western blot analysis of protein expression. Glyceraldehyde 3‐phosphate dehydrogenase (GAPDH) is used as loading control. G) VEGF secretion in the culture medium of MCF‐7 cells 24 h after treatments assessed by ELISA assay. Data are presented as box plots (center line, median; box limits, upper and lower quartiles; whiskers, SD; filled square points, mean; circle points, data).

As known, free nucleic acids can hardly be internalized by cells.^[^ ^14^ ^]^ As shown in confocal laser scanning microscope (CLSM) image of MCF‐7 cells after incubation with Cy7 labeled anti21 for 4 h, no distinguishable fluorescence is observed (**Figure** **2**A). Anti21 loaded with DNA nanoclews and FNCs are both effectively internalized, which can also be quantitatively characterized with flow cytometry (Figure 2B). As a negative control, conventional zwitterionic polymer PCBMA coating significantly inhibits the celluar uptake of DNA nanoclews, suggesting that fluorination is crucial for improving the cellular internalization.^[^ ^23^ ^]^ Since endosomal escape is a key step for cytosolic delivery of genetic drugs,^[^ ^24^ ^]^ we further study the intracellular distribution of DNA nanoclews and FNCs by co‐staining the treated MCF‐7 cells with Lysotracker Green. DNA nanoclews are trapped in lysosomes, while fluorination facilitates escape of FNCs from acidic endosomes/lysosomes. It is reported that the improved cellular internalization and endosomal escape of fluorinated nanomaterials are both resulting from the hydrophobic and lipophobic nature of fluorine.^[^ ^11a,b^ ^]^ As shown in Figure S9 (Supporting Information), the intracellular fate of DNA nanoclews relies on the hydrophobicity and lipophobicity of polymer coating.

Next, the NIR light triggered oligonucleotide release behavior of FNCs in the cytoplasm is investigated with two‐plex NIR fluorescence imaging. Anti21 is labeled with Cy7, a cyanine dye emitting fluorescence in NIR‐I (800–900 nm) and NIR‐IIa region (1000–1300 nm),^[^ ^25^ ^]^ and FNCs are labeled with NIR‐IIb emitting CSQDs. Therefore, the cytosolic release of anti21 can be tracked by fluorescence imaging from the two channels. Fluorescence signals of anti21 and CSQDs in the absence of NIR laser illumination are largely overlapped with a Pearson correlation coefficient (PCC) of 0.83 (Figure 2C). After laser illumination, photothermal effect induces dissociation of hydrogen bonding and anti21 and CSQDs are both released from FNCs, leading to separation of fluorescence signals from the two channels. We also compare the release of anti21 from a polycationic vector, hyperbranched polyamidoamine (PAMAM).^[^ ^26^ ^]^ Although PAMAM realizes high endocytosis efficiency, anti21 can hardly detach from PAMAM vector due to the strong electrostatic interactions.

As mentioned above, FNCs can effectively be internalized by cells and release anti21 in the cytoplasm upon laser irradiation (Figure 2D). Anti21 can subsequently bind to miR‐21 and downregulate the expression of miR‐21. The miRNA expression regulation of anti21 loaded with FNCs was examined by quantitative real‐time polymerase chain reaction (qRT‐PCR). FNCs without anti21 loading show no obvious disruption to miR‐21 expression regardless of NIR light illumination (Figure 2E), indicating no interference of both vectors and photothermal effect on gene expression. Anti21‐loaded FNCs effectively suppress miR‐21 expressions to 9.2% upon laser irradiation, while the silence efficiency in the absence of laser is negligible. Polycationic PAMAM as vectors, anti21 only silenced 45.5% of the miR‐21 expression. The high transfection efficiency of FNC plus NIR light makes it an ideal platform for on‐demand cytosolic delivery of oligonucleotides.

NIR light triggered silencing of miR‐21 leads to expression inhibition of its downstream proteins, including β‐catenin, phosphorylated signal transducer and activator of transcription 3 (pSTAT3) and hypoxia inducible factor 1‐α (HIF1‐α), and the downregulation of these transcription factor levels will finally suppress the expression of VEGF.^[^ ^27^ ^]^ As indicated by western‐blot analysis, NIR laser illumination significantly decreases the expression levels of β‐catenin, pSTAT3, HIF1‐α and VEGF in FNC‐incubated cells (Figure 2F; Figure S10, Supporting Information). Enzyme‐linked immunosorbent assay (ELISA) indicates that the secretion of VEGF also reduces 90% after treatment (Figure 2G). VEGF is a well‐known key signal protein in the angiogenesis of solid tumors,^[^ ^28^ ^]^ and thus the effective downregulation of VEGF secretion associated with photothermal triggered cytosolic release can be applied to suppress tumor angiogenesis and abrogate cancer.

### 2.3 Circulation, Biodistribution and In Vivo Delivery

**Figure 3:**
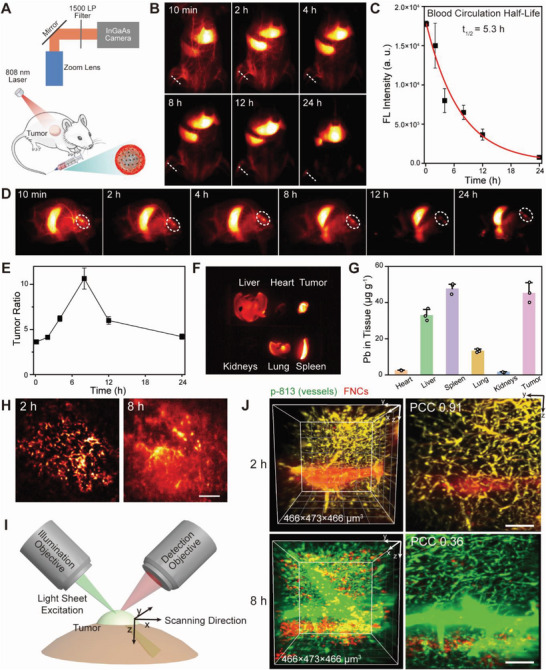
A) NIR‐IIb wide field imaging setup. B) NIR‐IIb fluorescence images of nude mice after tail‐vein injected with FNCs. C) Signal intensity of mouse femoral artery in the images are plotted as a function of time. The half‐life of FNCs blood circulation is estimated to be ≈5.3 h. D) NIR‐IIb images of the left flank, showing FNCs accumulating in the tumor. E) T/NT ratios of FNCs in tumor are plotted as a function of time. Mice treated with FNCs are sacrificed at 8 h post injection. The main organs and tumor are collected. NIR‐IIb imaging F) and ICP‐OES G) measurements both reveal the biodistribution of FNCs mainly in tumor, liver and spleen. Means ± SD in all graphs are from three independent measurements. H) High magnification imaging of the tumor at 2 h (left) and 8 h (right) post administration. Scale bar, 1 mm. I) Scheme of in vivo NIR‐II oblique LSM showing positions of illumination objective, detection objective and the tumor. J) Left: reconstructed 3D LSM images of the tumor 2 h (up) and 8 h (bottom) after FNC administration. Right: maximum intensity projections within a thickness of 100 µm along the x direction. White bars indicate 100 µm.

Zwitterionic polymer coating are widely reported to prolong blood circulation time of nanomedicine due to superhydration ability and neutral electrical charge.^[^ ^18b^ ^]^ Taking advantage of NIR‐IIb emitting CSQDs, the blood circulation behavior of FNCs can be readily explored using fluorescence imaging. Fluorescence imaging in the NIR‐IIb window is benefit from greatly reduced light scattering, affording deep tissue penetration with diminished background interference, and thus has drawn much interests for dynamic and noninvasive investigation of pharmacokinetics and biodistribution.^[^ ^20^, ^29^ ^]^ Nude mice bearing subcutaneous MCF‐7 tumor are intravenously injected with FNCs and then subjected to in vivo imaging at different time points. The NIR‐IIb imaging is conducted with a wide‐field setup equipped with an InGaAs charge coupled device camera, collecting emission signals under 808 nm excitation (**Figure** **3**A). In the analysis of blood samples after administration, no free CSQD can be detected in the serum, proving the stability of FNCs during circulation (Figure S11, Supporting Information). In this case, fluorescence intensity of vasculatures demonstrates blood circulation behavior of FNCs. As calculated, the blood circulation half‐life (t_1/2_) of FNCs is ≈5.3 h (Figure 3B,C). It can be intuitively figured out from the imaging results that FNCs primarily accumulate in tumor, spleen and liver after administration. Owing to passive accumulation through enhanced permeability and retention effect,^[^ ^30^ ^]^ the tumor to normal tissue fluorescence signal ratio (T/NT) increases sharply during the initial hours and peaks at 8 h post injection, suggesting most abundant enrichment (Figure 3D,E). Ex vivo imaging and quantification analysis of Pb elements with inductively coupled plasma optical emission spectrometer (ICP‐OES) in major viscera (heart, liver, spleen, lung and kidneys) and tumor tissues also confirm accumulation in tumor, liver and spleen, with little retention of FNCs in other organs (Figure 3F,G).

When zooming in to the tumor, the distribution of FNCs can be observed (Figure 3H): at 2 h post injection, most FNCs are circulating in the vessels and fluorescence signals resolve the tortuous tumor vasculature. At 8 h, signals diffused in the tumor region, indicating FNCs extravasation and internalization by cancer cells. The deep penetrated imaging in NIR‐IIb also leads to the overlap of fluorescence signals from various depth, making it difficult to acquire the volume spatial distribution of FNCs at the tumor site. NIR‐LSM enables noninvasive in vivo optical sectioning at cellular resolution in mammals.^[^ ^13^ ^]^ Therefore, we perform volumetric imaging of tumor through intact skin in live mice using a home‐built 45^o^ oblique NIR‐LSM (Figure 3I). A NIR nanofluorophore (p‐813), prepared by encapsulating a commercial hydrophobic NIR dye, IR813, in an amphiphilic polymer, polystyrene‐graft‐poly(ethylene glycol) (PS‐g‐PEG),^[^ ^29a^ ^]^ is injected via tail vein 5 min prior to imaging, so that circulating p‐813 can be used to label vasculatures. The fluorescence emission peak of p‐813 located at 872 nm upon excited with 808 nm laser, with a tail extended to NIR‐IIa region (Figure S12, Supporting Information). In this case, the whole delivery process of FNCs, including circulation, extravasation, penetration and endocytosis inside tumor can be visualized directly with two‐plex NIR imaging. The optical sectioning with LSM is conducted at various time points after FNC administration and the images acquired are reconstructed to get a 3D view. At 2 h, most fluorescence signals from two channels are overlapped, and there are some NIR‐IIb signals diffused around the blood vessels (Figure 3J). From these results we can infer that at this time, a large quantity of FNCs is in circulation, with some extravasated from vessels and penetrated into tumor tissues. At 8 h, signals from the two channels fully separated, indicating FNCs cleared out from vessels. And also, the diffused NIR‐IIb signals disappear and some bright spots in NIR‐IIb channels are observed, suggesting FNCs internalized by cancer cells (Figure 3I).^[^ ^31^ ^]^ Hence, 8 h after injection can be a good timing for laser irradiation to trigger cytosolic release of loaded oligonucleotides. Utilizing NIR‐IIb emitting CSQDs and NIR‐LSM, we achieve volumetric visualizing gene vectors entry into cancer cells in a live mammal noninvasively for the first time. This strategy can be extremely useful for pre‐clinic research in anti‐tumor nanomedicine and advancement of novel oncotherapy modalities.

### 2.4 NIR Light Activated Anti‐Angiogenesis Therapy

**Figure 4:**
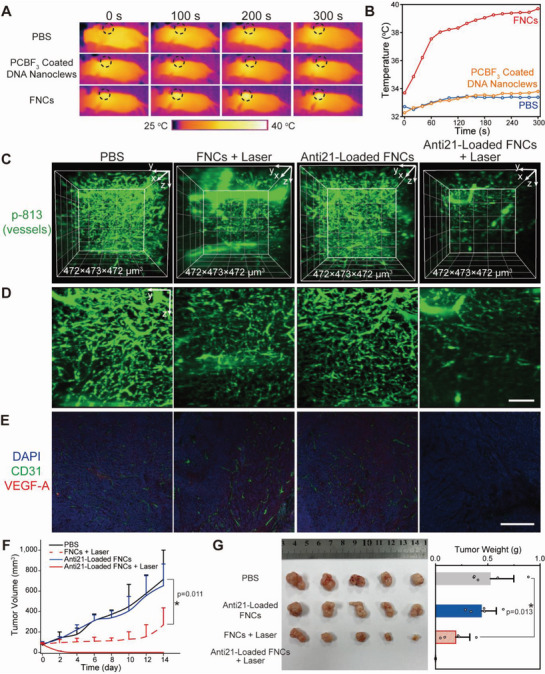
A) Thermal images of PBS, CSQD‐free PCBF_3_ coated DNA nanoclews and FNCs treated mice during laser illumination (808 nm, 5 min, 0.33 W cm^−2^). B) Heat curves of tumors upon laser illumination as a function of time. C) 3D noninvasive microscopic angiography in the tumor after treatments, obtained with NIR‐LSM for optical sectioning and p‐813 as contrast agents. D) Maximum intensity x projections for a 100 µm thick volume. White bars indicate 100 µm. E) Immunostaining of tumor sections 24 h after treatments. Scale bar indicates 0.5 mm. F) Tumor growth curves of MCF‐7 tumor bearing mice after different treatments. *, *p* < 0.05. G) Image (left) and weights (right) of tumors after excision from each group.

Being aware of the time point at which FNCs are uptaken, we then perform NIR light illumination with an 808 nm laser at the tumor sites for 300 s. Thermal imaging with an infrared thermal camera is used to record real‐time temperature during the treatment (**Figure** **4**A,B). The thermal images and heating temperature curves show no obvious local temperature rise in phosphate buffered saline (PBS) and CSQD‐free PCBF_3_ coated DNA nanoclew treated mice, while for FNC group, the temperature increases 5–6 °C to ca. 39 °C. The local heat generated during laser irradiation will trigger FNC deconstruction and cargo release, without any damage to periphery normal cells and tissues.

One day later, in vivo volumetric angiography in the tumor is conducted using NIR‐LSM for optical sectioning and p‐813 as contrast agent. The 3D imaging results show that angiogenesis in tumors treated with PBS or anti21‐loaded FNCs is not disrupted, suggesting negligible regulation effect without oligonucleotide release (Figure 4C,D). Extravasation of p‐813 from vessels in the two laser treated groups can be observed, which possibly results from local edema induced by the mild hyperthermia. Vascular growth inside FNC treated tumor is significantly inhibited after laser irradiation. Angiogenesis can also be examined by immunofluorescent staining of tumor tissue sections, showing fewer micro‐vessels (labeled with CD31 antibody) in the FNC plus laser group than the other groups (Figure 4E). Expressions of miR‐21 and its downstream proteins (β‐catenin, pSTAT3, HIF1‐α and VEGF) are also determined by qRT‐PCR (Figure S13, Supporting Information) and the photomicrographs of immunofluorescence, respectively (Figure 4E; Figure S14, Supporting Information). Expressions of miR‐21 and all the proteins are downregulated, demonstrating the in vivo release of antisense oligonucleotides and silencing of miR‐21 upon laser illumination. The results above prove NIR light can activate anti‐angiogenesis effect of FNCs by triggering cytosolic release of loaded anti21.

Owing to the suppression of vasculature formation, FNC treated tumors tend to necrosis soon after laser illumination. A single injection of FNCs plus laser irradiation completely ablate the tumor and only a thin scar is left at the tumor site two weeks later (Figure 4F; Figure S15, Supporting Information). On the contrary, tumors treated with PBS or anti21‐loaded FNCs grow rapidly, suggesting little therapeutic effect without cargo release. FNCs without anti21 loading also exhibit tumor growth inhibition after laser intervention, which is related to the mild hyperthermia generated from photothermal effect of FNCs. Whereas, lack of long‐term inhibition effect of gene silencing leads to recurrence of tumor. This indicates the tumor ablation of anti21‐loaded FNCs plus laser is actually resulting from a photothermal‐augmented gene therapy. At the 14^th^ day after injection, all tumor‐bearing mice are sacrificed and the tumors are dissected, weighed and imaged (Figure 4G). To explore the possible side effects, body weight of mice is simultaneously recorded every other day. No obvious body weight fluctuation occurs, indicating the treatments cause no severe systemic toxicity (Figure S16, Supporting Information). Histopathological examination is conducted to further ensure safety of the therapy. Hematoxylin and eosin (HE) stained sections of major organs show no signs of damage or other symptoms (Figure S17, Supporting Information). These data demonstrate that anti21‐loaded FNCs, possessing NIR light activatable anti‐angiogenesis activity with low systemic toxicity, can act as a hopeful nanomedicine for miRNA regulation in oncotherapy.

## 3 Conclusion

In the present work, we develop an approach to deliver oligonucleotides by means of encapsulating in a DNA nanoclews through Watson‐Crick hydrogen bonds, followed by fluorinated zwitterionic polymer coating. Further incorporating quantum dots provides the vector with NIR‐IIb fluorescence and NIR light triggered release ability. The FNC vector can deliver oligonucleotides to cancer cells systemically and realizes on‐demand cytosolic release of the cargo with high transfection efficiency. Taking advantage of the NIR‐IIb emission, we achieve noninvasive volumetric imaging of the gene vector entering into cancer cells from circulation. Loaded by FNC vector, an oligonucleotide, anti21, can effectively silence the target miRNA when activated with NIR light, and suppress tumor angiogenesis, leading to complete ablation of cancer. The excellent therapeutic efficacy proves the great potential of FNCs as an oligonucleotide delivery platform, broadening horizons for the design of novel gene vectors.

## 4 Experimental Section

### Preparation and Characterization of DNA Nanoclews

DNA nanoclews were prepared with RCA as previously described.^[^ ^9a^ ^]^ In brief, ssDNA template (Sangon Biotech) was cyclized into a circular ssDNA template with CircLigase II ssDNA ligase (CL9021K, Epicentre) according to the manufacturer's instructions. Then the cyclized template was treated with Exonulease I (C610019, Sangon Biotech) to eliminate any residue linear ssDNA. After deactivating enzymes by heating, the resultant cyclized ssDNA template was hybridized with primer and then Bst 2.0 DNA polymerase (M0537S, New England Biolabs) was added. RCA was performed at 60 °C for 17 h and then the polymerase was deactivated by heating. The obtained DNA nanoclews were purified using an Amicon Ultra centrifugal filter with a cutoff molecular weight (MWCO) of 50 kDa thrice with TE buffer (T1120, Solarbio). Concentration of DNA nanoclews was characterized with the UV absorbance at 260 nm using NP80, Implen. Particle size and zeta potential were measured by dynamic light scattering with Brookhaven 90Plus Zeta. TEM samples were prepared by dropping DNA nanoclews onto copper grid and negatively staining with 1% phosphotungstic acid, which was subsequently imaged with TEM (JEM‐1400, JEOL).

### Preparation and Characterization of FNCs

N‐hydroxy succinimide ester of Cy7 (Cy7‐NHS, OK‐F‐13106, Okeanos) was conjugated to amine‐terminated anti21 sequence (Sangon Biotech). Next, DNA nanoclews, anti21‐Cy7 and BAAPy were mixed with a mass ratio of 8:2:1 and hybridized in a heating‐cooling cycle. The anti21‐loaded DNA nanoclews were purified with centrifugal filter (MWCO 50 kDa) and dispersed in a carbonate buffer (50 mm, pH 8.5) at 1 mg mL^−1^ for further use. The amount of anti21 in DNA nanoclews was calculated by subtracting the mass of anti21 in the filtrate from the total mass of anti21 in the initial solution, quantified by NIR‐IIa fluorescence of anti21‐Cy7 using a pre‐established calibration curve. The fluorescence analysis was conducted using a wide‐field NIR imaging setup with 660 nm laser excitation (MDL‐III‐660D, Cnilaser) and 1000 nm long pass filter (FELH1000, Thorlabs). Then CBF_3_ (5 mg), methacrylamide modified CSQDs (3 mg) and (NH_4_)_2_S_2_O_8_ (2 mg) dissolved in 0.5 mL deionized water was added to 5 mL abovementioned DNA nanoclew dispersion. The vial was sealed and degassed with nitrogen thrice. N,N,N’,N’‐tetramethylethylenediamine (4 µL) were injected into the mixture to initiate free radical polymerization, which proceeded for 1 h at room temperature. Finally, FNCs were obtained by centrifugation (10 000 rpm, 25 min) and washed with PBS buffer thrice.

For stability study, free anti21, anti21‐loaded DNA nanoclews and anti21‐loaded FNCs were incubated with RNase A (1 mg mL^−1^, R1030, Solarbio) or fetal bovine serum (FBS, 10%) for 2 h at 37 °C and then heated to 95 °C to release the oligonucleotides. Electrophoresis was carried out in 2 w/v % agarose gel. Owing to the NIR‐IIa fluorescence of anti21‐Cy7, the agarose gel was directly imaged using the wide‐field setup equipped with an InGaAs camera (NIRvana HS, Princeton Instruments). Schematic and photograph of the setup were shown in Figure S18 (Supporting Information). The anti‐fouling ability was evaluated by mixing FNCs (1 mg mL^−1^) with HSA (2 mg mL^−1^) in PBS buffer and the colloid data were collected every day for a week.

### NIR Light Triggered Release

The absorption spectrum of FNCs was obtained with an UV–vis spectrometer (Evolution 300, Thermo Scientific). The emission spectra of anti21‐Cy7 and p813 in the far red and NIR‐I region was measured with a fluorescence spectrometer (Lumina, Thermo Scientific). A home‐built setup was used to measure the fluorescence emission spectra in the region of 900–1700 nm using a fiber spectrometer (NIR17S, Idea Optics) with 25 µm slit. The absolute quantum yield measurement was performed as previously described^[^ ^20^ ^]^ using an integrating sphere (IS210C, Thorlabs) equipped with an InGaAs photodiode (SM05PD5A, Thorlabs) as detector and an amplifier (PDA200C, Thorlabs). The PTC effect was evaluated by illuminating CSQD or FNC dispersion (2 mg mL^−1^, 100 µL) in a 96‐well plate with 808 nm continuous wave laser (fluence: 0.33 W cm^−2^, duration: 0–300 s, MDL‐III‐808, Cnilaser). The temperature was measured using a thermal camera (E8‐XT, FLIR) and the PTC efficiency was calculated using the cooling curve as previously reported.^[^ ^32^ ^]^ To investigate the photothermal‐triggered release of anti21, a dispersion of FNCs in PBS was shaked at 37 °C and illuminated by 808 nm laser. Five minutes after illumination, the dispersion was filtrated with a centrifugal filter (MWCO 50 kDa) and the amount of anti21‐Cy7 in the filtrate was determined with NIR‐IIa fluorescence intensity.

### Cytotoxicity and Cellular Uptake Assay

MCF‐7 cells were purchased from Cells Bank of Chinese Academy of Sciences and cultured in a DMEM medium supplemented with FBS (10%), L‐glutamine (2 mm), penicillin (100 U mL^−1^), and streptomycin (100 U mL^−1^) at 37 °C and 5% CO_2_. The cells were seeded in a 96‐well plate at a density of 5 × 10^3^ viable cells per well and incubated for 24 h to allow cell attachment. Then the cells were incubated with free anti21, DNA nanoclews or FNCs at indicated concentrations, respectively. Three hours later, some of the wells were illuminated by 808 nm laser. After incubation for 48 h, the culture media were aspirated and CCK8 agent, diluted with DMEM (10 v/v %), was added to the wells. The 96‐well plate was subjected to a multi‐well scanning spectrophotometer (Multiskan GO, Thermofisher) and absorbance value of each well at 490 nm was recorded. The final relative cell viability was obtained by comparting absorbance values of control and experimental wells.

### Cellular Uptake Assay was Conducted as Follows

MCF‐7 cells were seeded in 24‐well plates and cultured with free anti21‐Cy7 or vectors loaded with anti21‐Cy7 for 3 h. Then the cells were trypsinized, resuspended in the medium and washed with PBS. The intracellular fluorescence was determined using a flow cytometer (LSRFortessa X‐20, BD).

To investigate the localization of FNCs, MCF‐7 cells were seeded in glass bottom dishes and cultured in FNCs containing medium at 37 °C for 3 h, followed by washed with PBS thrice to fully remove nonspecifically adsorbed vectors. And then, the cells were stained with 250 nm of Lysotracker Green DND‐26 (Ex 504 nm, Em 511 nm, Invitrogen) for 20 min and 5 µg mL^−1^ Hoechst 33 342 (Ex 345 nm, Em 478 nm, Solarbio) for another 10 min. After the staining solution was removed, the cells were rinsed with PBS thrice, fixed with 2% paraformaldehyde for 15 min at 4 °C and subjected to CLSM (A1, Nikon). Hoechst, Lysotracker and Cy7 were excited by 404 nm (3% power), 488 nm (20% power), and 638 nm (20% power) laser, respectively, and their emission windows were set as 430–475 nm, 500–550 nm, and 663–738 nm, respectively. All images were captured under the same instrumental settings and analyzed with ImageJ software.

### Cytosolic Release

Cells treated with FNCs were imaged using a home‐built NIR microscope (shown in Figure S19, Supporting Information). Cy7 was excited with 660 nm laser (60 mW cm^−2^) and the emission photons in the 1000–1300 nm region was collected with a NIR camera (NIRvana HS, Princeton Instruments) with exposure time of 100 ms. CSQDs were excited with 808 nm laser (60 mW cm^−2^) and the emission photons were allowed to pass through a 1500 nm longpass filter so that fluorescence in the 1500–1700 nm region could be collected by the NIR camera (exposure time: 100 ms). The Coloc 2 plugin of ImageJ was used to calculate the PCC. NIR illumination was applied with 808 nm laser at a power density of 0.33 W cm^−2^ for 300 s.

Expression level of miR‐21 after laser illumination was determined with qRT‐PCR. MCF‐7 cells were seeded on 6‐well plates for 24 h to achieve 70% confluence. Then FNCs were added and incubated at 37 °C for 3 h. After laser illumination, cells were further incubated for 24 h. The total RNA was extracted with Trizol according to the manufacturer's instructions. Complementary DNA (cDNA) was synthesized using All‐in‐One miRNA qRT‐PCR detection kit (QP116, Geneopoeia) and PCR reactions were performed with StepOnePlus RT‐PCR systems (Applied Biosystems). Relative quantification of miR‐21 expression was conducted using amplification efficiencies derived from cDNA standard curves and data were shown as fold change.

For western blot analysis, cells were treated as mentioned above. Cells were then lysed with RIPA Lysis Buffer (P0013, Beyotime). The total protein was quantified using an Enhanced BCA Protein Assay Kit (P0010, Beyotime). The protein lysates were separated by SDS‐PAGE and then transferred to polyvinylidene fluoride membranes. The membranes were incubated with primary antibodies against HIF 1‐α, β‐catenin, pSTAT3 and VEGF (1:1000 dilution, AF1009, AF6266, AF3293, AF5131, Affinity), followed by incubation with an HRP‐conjugated secondary antibody (1:5000 dilution, SA00001, Proteintech). GAPDH (1:1000 dilution, AP0066, Bioworld) was set as a loading control. The membrane was imaged with a chemoluminescence imaging system (5200CE, Tanon). Secretion levels of VEGF in the cell culture media were further evaluated with ELISA. The culture media were collected and quantified using a human VEGF ELISA kit (SEKH‐0052, Solarbio) based on the manufacturer's protocol.

### Animals and Tumor Model

Female BALB/c nude mice (4 weeks old, ca. 20 g body weight) were purchased from Risemice Biotechnology. Animal care and handling procedures were in agreement with the guidelines evaluated and approved by the ethics committee of Southern Medical University (SYXK(粤)2016‐0167). A rodent anesthesia machine with 10 L min^−1^ air flow mixed with 2.5% isoflurane was used to anesthetize the mice and the anesthetic was kept delivering with a nose cone during tumor inoculation and imaging. For tumor model construction, MCF‐7 tumors were inoculated by subcutaneous injection of 2 × 10^6^ cells in 100 µL of serum‐free DMEM medium onto the back of each mouse. The tumor sizes were measured by a caliper every other day and the tumor volume was calculated as (tumor length) × (tumor width)^2^/2. The mice were randomly divided into 4 groups and used for imaging and treatment when tumor volume reached 50 mm^3^ (≈4–5 d post inoculation).

### Circulation and Biodistribution

To investigate circulation time and biodistribution, wide field imaging was applied to mice intravenously injected with FNCs using an InGaAs camera (NIRvana HS, Princeton Instruments) equipped with a zoom lens (1‐50502‐IR, Navitar). The excitation light was provided by an 808 nm laser through a multi‐mode optical fiber (F‐MSC‐C‐1SMA, Newport). The average excitation power density at the imaging plane was 60 mW cm^−2^. The emission fluorescence was allowed to pass through a 1500 nm longpass filter (FELH1500, Thorlabs) to ensure that photons of 1500–1700 nm wavelength were collected. The upper bound at 1700 nm was determined by the sensitivity profile of the InGaAs camera. For ex vivo fluorescent imaging and biodistribution assays, the mice were sacrificed at 24 h post injection by cervical dislocation. Tumors and major organs were dissected, washed with cold saline and subjected to wide field imaging. Pb uptake in the tumor and major organs was measured with ICP‐OES (iCAP Pro, Thermo Fisher). A volume of 60% nitric acid was added to each tissue homogenate sample and incubated for 24 h at 60 °C. The solutions were centrifuged at 10 000 rpm for 25 min and the supernatant was diluted ten‐fold with 2% nitric acid. The concentration of Pb^2+^ was measured by ICP‐OES and calculated as microgram of Pb content per gram of tissue (µg Pb/g tissue).

### Volumetric Imaging

The details of NIR‐LSM setup was described previously.^[^ ^13^ ^]^ This home built LSM employed 808 nm laser cylindrically focused into static light sheets for optical sectioning and an InGaAs camera (NIRvana HS, Princeton Instruments) for orthogonally fluorescence detection in NIR‐IIa and NIR‐IIb (setup detailed in Figure S20, Supporting Information). The illumination objective and the detection objective were arranged 45^o^ to the sample surface. Planar movement of the mouse was controlled with a motorized linear stage (9065‐XYZ‐PPN‐M, Newport). A 4X illumination objective (NA = 0.13, N4X‐PF, Nikon) and a 10X imaging objective (NA = 0.3, N10X‐PF, Nikon) were used in this work. The recorded original images were 45^o^ to the horizontal direction. These images were transformed to xyz dimensions in ImageJ/Fiji with affine functions. In a typical experiment, the p‐813 and FNCs were both excited with 808 nm laser and the scanning step was 5 µm. Filters of 1000 nm longpass (FELH1000, Thorlabs) and 1300 nm shortpass (89‐676, Edmund Optics) were used to collect the fluorescence signal of p‐813 and a 1500 nm longpass filter (FELH1500, Thorlabs) was used to collect fluorescence signal of FNCs.

### Anti‐Angiogenesis Therapy

Twenty‐four hours after administration of FNCs, tumors were exposed to 808 nm laser at a power density of 0.33 W cm^−2^ for 5 min. Temperature of tumor site was recorded by a thermal camera (E8‐XT, FLIR). Another 24 h later, three mice in each group were euthanized and the tumors were dissected. Subsequently, the tissues were embedded in optimal cutting temperature compound and placed at −20 °C for 24 h. A frozen section machine was used to obtain applicable freezing slice (thickness of 10 µm). These slices were immunostained with primary rabbit antibodies against HIF 1‐α, β‐catenin, pSTAT3 and VEGF (1:100 dilution, AF1009, AF6266, AF3293, AF5131, Affinity), respectively, followed by incubation with an AF568‐conjugated Anti‐Rabbit antibody (1:500 dilution, A10042, Invitrogen). Anti‐VEGF‐stained slices were co‐stained with goat anti‐CD31 (10 µg mL^−1^, AF3628, R&D Systems), whose secondary antibody was FITC‐conjugated anti‐goat (1:50, SA00003‐3, Proteintech). Images of all stained slices were acquired with CLSM (A1, Nikon) with the same settings.

Two weeks later, all mice were imaged and sacrificed. All tumors were dissected and weighed, and major viscera were harvested and fixed in a 10% formalin solution. For histopathological tests, the tissue samples were embedded in paraffin blocks, sectioned into 5 µm slices and mounted onto the glass slides. After HE staining, the sections were examined by a digital microscope (Eclipse Ni‐U, Nikon).

## Conflict of Interest

The authors declare no conflict of interest.

## Supporting information

Supporting Information

## Data Availability

The data that support the findings of this study are available from the corresponding author upon reasonable request.
