# Supplementary material for: Fluoropolymer Coated DNA Nanoclews for Volumetric Visualization of Oligonucleotides Delivery and Near Infrared Light Activated Anti‐Angiogenic Oncotherapy
Source: Adv Sci (Weinh). 2023 Sep 28;10(32):2304633. doi: 10.1002/advs.202304633 (PMC10646232; doi:10.1002/advs.202304633)
Supplement: Supplementary file 1 — Supporting Information [file ADVS-10-2304633-s001.pdf]

ADVANCED SCIENCE
Open Access

Supporting Information

for *Adv. Sci.*, DOI 10.1002/advs.202304633

Fluoropolymer Coated DNA Nanoclews for Volumetric Visualization of Oligonucleotides Delivery and Near Infrared Light Activated Anti-Angiogenic Oncotherapy

Peng Zhang, Ranran Guo, Haiting Zhang, Wuli Yang and Ye Tian**

Supporting Information

Fluoropolymer Coated DNA Nanoclews for Volumetric Visualization of Oligonucleotides Delivery and Near Infrared Light Activated Anti-Angiogenic Oncotherapy

Peng Zhang, Ranran Guo, Haiting Zhang, Wuli Yang and Ye Tian**

1. Conjugation of Cy7 with Anti21

Amine-terminated anti21 (10 µg) was dissolved in a buffer of 4-morpholineethanesulfonic acid (MES, 0.2 mL, 10 mM, pH 8.5). Cy7-NHS (1.1 µg) dissolved in dimethyl sulfoxide (20 µL) was added to the above solution and the mixture was shaken for 3 h in dark. The resultant anti21-Cy7 was washed by centrifugal filter (MWCO 3 kDa) thrice against RNase-free PBS buffer to remove excess dye and generated NHS.

2. Synthesis of BAAPy

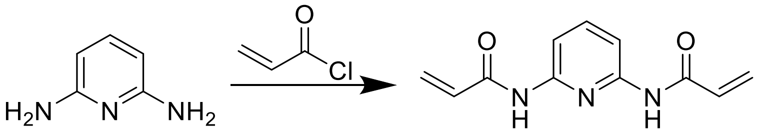

BAAPy was synthesized according to a published protocol.[1] 2,6-Diaminopyridine (2.18 g, 20 mmol, 1 eq) were dissolved in tetrahydrofuran (THF, 100 mL) and trimethylamine (TEA, 7 mL, 50 mmol, 2.5 eq) was added. The mixture was bubbled with N_2 and cooled in an ice bath. Acryloyl chloride (3.6 mL, 44 mmol, 2.2 eq) diluted with THF (10 mL) was added dropwise to the vigorously stirred mixture mentioned above. After completely addition of acryloyl chloride, the reaction mixture was warmed to room temperature and stirred for another 2 h. Water (50 mL) was added to quench the reaction and the layers were separated. The aqueous phase was washed with $CHCl_3$ (100 mL). The organic layers were combined, washed with saturated $NaHCO_3$ (40 mL) and water in sequence and concentrated under vacuum. The crude product was recrystallized from toluene to get BAAPy as a yellow solid (2.17 g, 50% yield). ^{1}H-NMR (600 MHz, DMSO-d_6, δ ppm): 10.31 (br, 2H, -NH-), 7.86 (d, 2H, J = 7.8 Hz, Py-H), 7.79 (t,

1H, J = 7.9 Hz, Py-H), 6.65 (dd, 2H, J_1 = 17.0 Hz, J_2 = 10.1 Hz, =CH-), 6.31 (dd, 2H, J_1 = 17.0 Hz, J_2 = 1.8 Hz, $=CH_2$), 5.79 (dd, 2H, J_1 = 10.1 Hz, J_2 = 1.8 Hz, $=CH_2$).

3. Synthesis of CBF_3

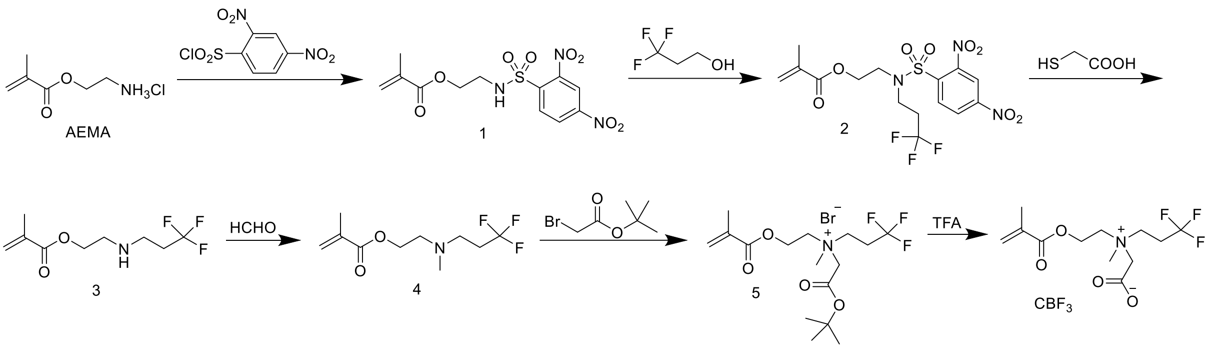

CBF_3 was synthesized as previously reported.[2] In brief, amine group of 2-aminoethyl methacrylate hydrochloride (AEMA) was firstly protected with 2,4-dinitrobenzensufonyl (DNs) group. Acidic sulfonamide could subsequently react with fluorinated alcohol in a Mitsunobu reaction. After deprotection with thiols, the secondary amine group was methylated in an Eschweiler-Clarke reaction. Nucleophilic substitution between the tertiary amine and tert-butyl bromoacetate followed by deprotection of carboxyl group yielded the final fluorinated zwitterionic product, CBF_3.

3.1 Synthesis of N-(2-methacryloyloxy ethyl)-2,4-dinitrobenzenesulfonamide (Compound 1)

AEMA (5.0 g, 27.2 mmol, 1 eq) was dissolved in THF (50 mL). The solution was cooled with an ice bath and TEA (7.5 mL, 54.4 mmol, 2 eq) was added. After 15 min stirring, DNsCl (8.7 g, 32.6 mmol, 1.2 eq) pre-dissolved in THF (10 mL) was added dropwise to the reaction mixture. The reaction mixture was then warmed to room temperature and stirred for 24 h. The reaction mixture was filtered and the filtrate was evaporated in vacuum. The crude product was purified by silica gel chromatography with a mixture of dichloromethane and hexane (4:1) as eluent, obtaining compound 1 as a yellow solid (8.6 g, yield 88%). ^{1}H-NMR (600 MHz, $CDCl_3$, δ ppm): 8.68 (d, 1H, J = 8.6 Hz, Ph-H), 8.56 (dd, 1H, J_1 = 8.6 Hz, J_2 = 2.2 Hz, Ph-H), 8.37 (d, 1H, J = 8.6 Hz, Ph-H), 6.05 (s, 1H), 5.77 (s, 1H), 5.64 (br, 1H, -NH-), 4.34 (t, 2H, J = 5.8 Hz, -O-CH_2-), 3.69 (td, 2H, J_1 = 10.6 Hz, J_2 = 5.8 Hz, -C$\underline{H}_2$-NH-), 1.89 (s, 3H, -CH_3).

3.2 Synthesis of N-(3,3,3-trifluoropropyl)-N-(2-methacryloyloxy ethyl)-2,4-dinitrobenzenesulfonamide (Compound 2)

Compound 1 (3.59 g, 10 mmol, 1 eq) was dissolved in THF (50 mL). To the ice-cooled solution, PPh_3 (5.25 g, 20 mmol, 2 eq) and 3,3,3-trifluoropropanol (1.32 mL, 15 mmol, 1.5 eq) was added. After 15 min of stirring, diethyl azodicarboxylate (3.15 mL, 20 mmol) in THF (10 mL) was added dropwise to the above solution. The reaction was warmed to room temperature

and continued for 24 h. Then hexane was added dropwise to the reaction mixture until the solution turned turbid. The mixture was kept at -20 °C for 12 h to allow precipitation of $O=PPh_3$ byproduct. After filtration and concentration in *vacuo*, the crude product was purified by silica gel chromatography (ethyl acetate: hexane=4:1), obtaining compound 2 as a yellow solid (4 g, yield 88%). ^{1}H-NMR (600 MHz, $CDCl_3$, δ ppm): 8.95 (d, 1H, J = 2.5 Hz, Ph-H), 8.77 (dd, 1H, J_1 = 11.2 Hz, J_2 = 2.5 Hz, Ph-H), 8.25 (d, 1H, J = 8.5 Hz, Ph-H), 6.02 (s, 1H), 5.57 (s, 1H), 4.29 (t, 2H, J = 5.2 Hz, -O-CH_2-), 3.69 (t, 2H, J = 5.2 Hz, -C$\underline{H}_2$-N-), 3.62 (t, 2H, J = 7.1 Hz, -C$\underline{H}_2$-N-), 2.49 (dt, 2H, J_1 = 20.4 Hz, J_2 = 10.2 Hz, -CH_2-CF_3), 1.88 (s, 3H, -CH_3).

3.3 Synthesis of 2-(3,3,3-trifluoropropyl amino) ethyl methacrylate (Compound 3)

Compound 2 (4 g, 8.8 mmol, 1 eq) was dissolved in CH_2Cl_2 (45 mL) and mercaptoacetic acid (0.8 mL, 11.4 mmol, 1.3 eq) and TEA (2.45 mL, 17.6 mmol, 2 eq) was added. The reaction mixture was stirred for 15 min at room temperature and the washed with NaOH aqueous solution (0.5 M, 45 mL). The organic layer was dried with $MgSO_4$, filtered and concentrated in *vacuo*. The crude product was purified by silica gel chromatography (ethyl acetate:hexane=4:1), obtaining compound 3 as yellow oil (1.57 g, 79% yield). ^{1}H-NMR (600 MHz, $CDCl_3$, δ ppm): 6.05 (s, 1H, =CH_2), 5.52 (s, 1H, =CH_2), 4.19 (t, 2H, J = 5.4 Hz, -O-CH_2), 2.87 (t, 2H, J = 5.7 Hz, -C$\underline{H}_2$-NH-), 2.85 (t, 2H, J = 5.7 Hz, -C$\underline{H}_2$-NH-), 2.24 (m, 2H, -CH_2-CF_3),1.89 (s, 3H, -CH_3), 1.37 (s, 1H, -NH-).

3.4 Synthesis of 2-(N-methyl-N-(3,3,3-trifluoropropyl)amino)ethyl methacrylate (Compound 4)

Formic acid (10 mL) and formalin (10 mL) were mixed and compound 3 (1.13 g, 5 mmol) were dissolved in the mixed solvent. The mixture was heated at 70 °C in a N_2 atmosphere for 48 h. After cooling to room temperature, the reaction mixture was added dropwise to an ice-cooled NaOH solution (2.5 M, 100 mL). Then the aqueous solution was extracted with CH_2Cl_2 (5×100 mL). The organic layer was combined and dried with $MgSO_4$. The crude product was obtained by vacuum concentration and used for the next step without further purification.

3.5 Synthesis of CBF_3

Compound 4 (1.2 g, 5 mmol, 1 eq) was dissolved in acetonitrile (10 mL) and tert-butyl bromoacetate (1.1 mL, 7.5 mmol, 1.5 eq) was added. The mixture was bubbled with N_2 and heated at 50 °C for 24 h. After cooling to room temperature, ice cold diethyl ether was added to the mixture. The precipitate was collected and dried under *vacuo* to obtain compound 5 as a white solid.

CH_2Cl_2 (5 mL) and trifluoroacetic acid (5 mL) were mixed and compound 5 (2.17 g, 5 mmol, 1 eq) were dissolved in the mixed solvent. After addition of triethylsilane (2 mL, 12.5 mmol, 2.5 eq) to eliminate tert-butyl cations, the reaction mixture was stirred for 2 h at room

temperature. The solvents were then evaporated and the crude product was washed with diethyl ether to obtain final product CBF_3 as white solid after dried in vacuum (1.29 g, 87% yield). ^{1}H-NMR (600 MHz, D_2O, δ ppm): 6.11 (s, 1H, $=CH_2$), 5.74 (s, 1H, $=CH_2$), 4.64 (t, 2H, J = 4.5 Hz, $-O-CH_2$), 4.36 (s, 2H, $-CH_2-CO-$), 3.91 (t, 2H, J = 5.7 Hz, $-CH_2-N-$), 3.88 (t, 2H, J = 5.7 Hz, $-CH_2-N-$), 3.33 (s, 3H, $-N-CH_3$), 2.92 (m, 2H, $-CH_2-CF_3$), 1.89 (s, 3H, $=C-CH_3$).

4. Preparation of methacrylamide modified CSQDs

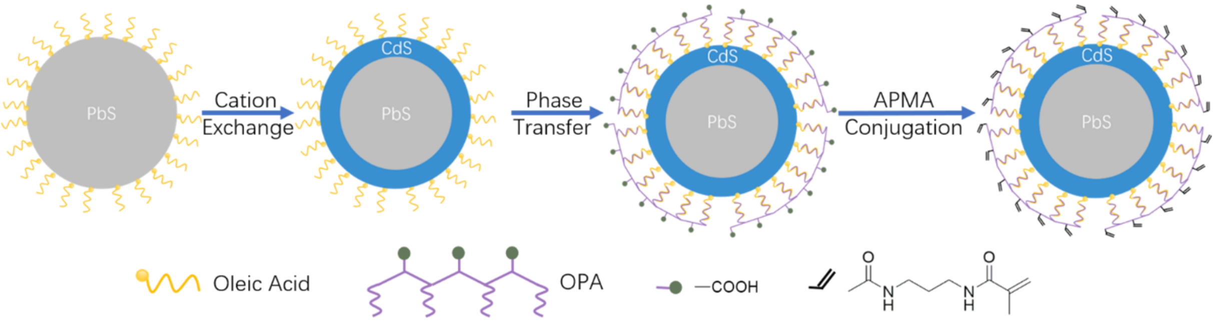

CSQDs were prepared as previously reported and then transferred to aqueous phase with oleyamine-branched polyacrylic acid (OPA).[3] N-(3-aminopropyl) methacrylamide hydrochloride (APMA) was then conjugated to OPA modified CSQDs through 1-(3-dimethylaminopropyl)-3-ethylcarbodiimide hydrochloride (EDC) chemistry.

4.1 Preparation of PbS QDs

$PbCl_2$ (834 mg, 3 mmol) was dissolved in oleylamine (7.5 mL) and the solution was degassed at 120 °C for 30 min. Then the vial was filled with nitrogen and heated to 160 °C. Sulfur (24 mg, 0.75 mmol) dissolved in oleylamine (2.25 mL) was injected into the above solution. After 30 min, the reaction was quenched by adding a mixed solution of hexane and ethanol (30 mL, 1:2). The QDs were collected by centrifugation and resuspended in hexane (10 mL). Oleic acid (20 mL) was added to precipitated the QDs, which was separated via centrifugation. The precipitation was repeated thrice and the PbS QDs were resuspended in 1-octadecene (ODE, 5 mL).

4.2 Cation Exchange to Prepare CSQDs

CdO (1.2 g, 9.2 mmol) were dispersed in ODE (20 mL) and oleic acid (8 mL) was added. The mixture was degassed and heated to 200 °C until CdO fully dissolved. Then the vial was filled with nitrogen and cooled to 100 °C. Previously prepared PbS QDs were injected into the system and the reaction was proceeded for 30 min. Hexane (5 mL) was added afterwards to quench the reaction. CSQDs were precipitated with ethanol and then redispersed in hexane for further use. The concentrations were determined gravimetrically.

4.3 Synthesis of OPA

OPA was synthesized with a modified procedure. Poly(acrylic acid) (0.9 g, Mw 1800) and N,N'-dicyclohexylcarbodiimide were dissolved in N,N-dimethylformamide (10 mL). Oleylamine (1.2 mL) was added dropwise into the system. The solution was stirred overnight and then HCl (0.5 M, 50 mL) was added. The precipitate was separated by centrifugation and extracted with $CHCl_3$ (5 mL). The organic solution was washed with HCl (1 M, 10 mL) and then dried with Na_2SO_4. The solvent was evaporated and OPA was obtained as a white solid. GPC characterization of OPA was shown in Figure S21A.

4.4 Phase Transfer of CSQDs

OPA (15 mg) was dissolved in $CHCl_3$ (2 mL), which was used to dispersed CSQDs (5 mg). The mixture was stirred at room temperature for 30 min and rotovaped. The residue was then dispersed in Na_2CO_3 solution (50 mM, 2 mL) under sonication. CSQDs were collected with a centrifugal filter (MWCO 10 kDa) and redispersed in MES buffer (0.1 M, pH 8.5).

4.5 Conjugation of APMA on CSQDs

Aqueous CSQDs (5 mg) were dispersed in MES buffer (20 mL) and EDC (10 mg) was added with stirring. APMA (0.7 mg) dissolved in MES buffer (0.5 mL) was then added to the above mixture, which was stirred overnight. The methacrylamide modified CSQDs was purified with centrifugal filter (MWCO 10 kDa) and redispersed in PBS for further use.

5. Transfection with hyperbranced PAMAM

5.1 Synthesis of hyperbranced PAMAM

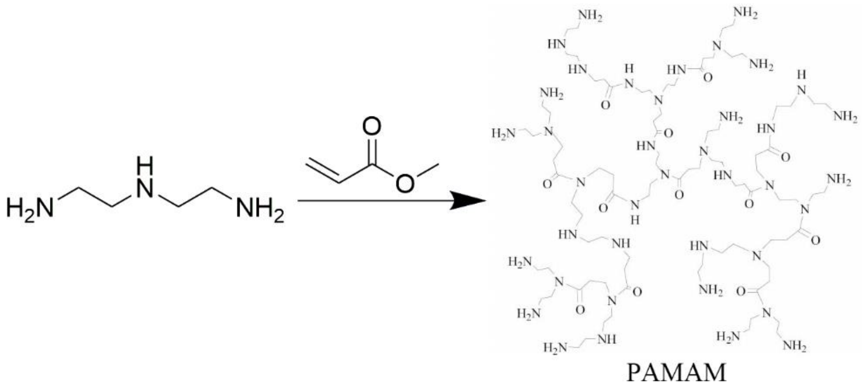

PAMAM

PAMAM was synthesized according to a previously reported method.[4] Diethylene triamine (20 g) was dissolved in methanol (30 mL) and methyl acrylate (20 g) in methanol (30 mL) was added dropwise to the above solution in a N_2 atmosphere. The mixture was stirred at room temperature for 72 h and distilled under vacuum to remove the solvent. Then the distillation temperature rose to 60 ^oC for 1 h, 80 ^oC for 1 h, 100 ^oC for 1.5 h, 120 ^oC for 1.5 h and 140 ^oC for 3 h. The crude product was dissolved in water (30 mL) and dialyzed for 3 d to eliminate monomers and oligomers. The final product was obtained by precipitation in acetone and dried under vacuum.

5.2 Transfection of Anti21

PAMAM and anti21-Cy7 were mixed with a N/P ratio (molar ratio between amine group of PAMAM and phosphate groups of oligonucleotides) of 10:1. The mixture was vortexed for 30 s and incubated at 37 ^{o}C for 30 min. The endocytosis and transfection efficiency of PAMAM-anti21 complex was determined using the same procedure as those of FNCs. The cytosolic release behavior was investigated by observing the fluorescence signal overlap of PAMAM and anti21-Cy7 with CLSM. It was not necessary to label PAMAM with fluorophores, since PAMAM emitted bright blue fluorescence when excited with UV laser.[5]

6. Preparation of p-813

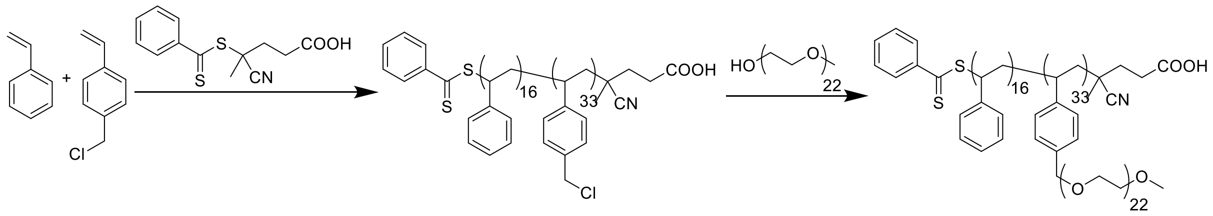

PS-g-PEG was synthesized as reported previously.[6] In brief, a gradient copolymer, p oly(styrene-co-vinylbenzyl chloride) was synthesized with reversible addition fragmentation transfer (RAFT) polymerization. Then methoxy-PEG (mPEG) was grafted onto the backbone via nucleophilic substitution between hydroxyl group of mPEG and benzyl chloride in the polymer. IR813 was encapsulated in the PS-g-PEG to obtain p-813.

6.1 Synthesis of Poly(Styrene-co-Vinylbenzyl Chloride)

Chain transfer agent, 4-cyano-4-(phenylcarbonothioylthio) pentanoic acid (28 mg, 0.1 mmol), and initiator azodiisobutyronitrile (4 mg, 0.02 mmol) were dissolve in 4-(chloromethyl) styrene (923 µL, 6.55 mmol) and styrene (374 µL, 3.26 mmol) was added. The solution was degassed by three freeze-pump-thaw cycles and heated at 90 ^{o}C for 20 h. After cooling to room temperature, CH_2Cl_2 was added to dissolve the crude product, which was then precipitated with 20-fold methanol. The precipitation was repeated thrice and the product was dried under vaccum. M_w = 7700 g mol^{-1}, PDI = 1.10 (Figure S21B).

6.2 Synthesis of PS-g-PEG

The copolymer (50 mg), mPEG (0.35 g, Mw 1000) and NaOH powder (20 mg) were placed in a Schlenk flask and degassed with N_2 thrice. Anhydrous THF (1.5 mL) was added and the reaction was proceeded at room temperature overnight. The mixture was filtrated to remove generated NaCl and residual NaOH, and the filtrate was evaporated. The crude product was dialyzed (MWCO 7 kDa) for 3 d and lyophilized to obtain the grafted polymer as a light yellow wax. M_w = 53000 g mol^{-1}, PDI = 1.88 (Figure S21B).

6.3 Encapsulation of IR813

IR813 tosylate (I863886, Macklin, 30 μg) and PS-g-PEG (10 mg) were dissolved in chloroform (100 μL). The solvent was evaporated at room temperature overnight. All the substances were reconstituted with PBS buffer (200 μL) under sonication for 10 min. The mixture was centrifuged at 8500 rpm for 30 min to remove precipitates, obtaining p-813.

7. Statistical Analysis

Unless otherwise stated, all data in this paper were expressed as mean result ± SD. For each representative experimental result, the number of successful independent experiments performed was indicated in the corresponding figure legend. Unpaired student's *t*-test was used for comparison between two testing groups and a probability (*p*) less than 0.05 was considered statistical significance. For imaging analysis, background was measured from a randomly selected area without vasculature or tumor. T/NT was the ratio of fluorescence signals in the whole tumor area over the randomly selected background. To reconstruct the optical sectioning images into 3D, Imaris 8.1 software was used for reconstruction and rendering 3D images from different viewing angles.

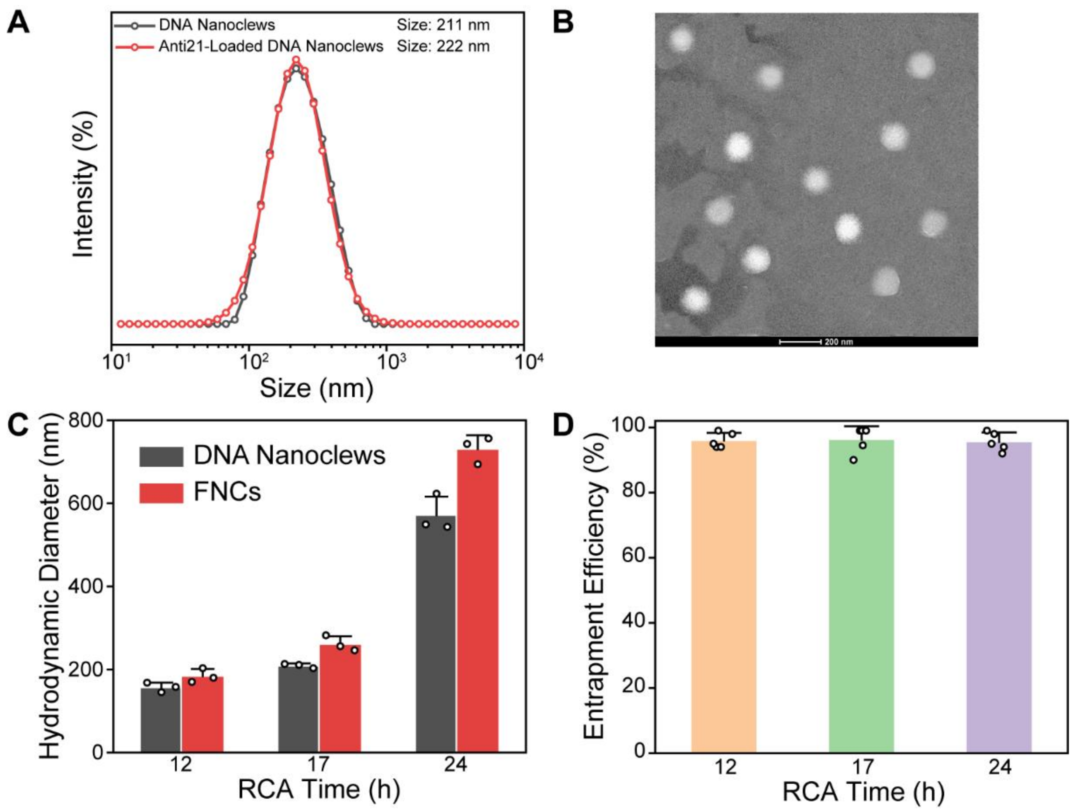

Figure S1. A) Size distribution of DNA nanoclews before and after anti21 loading. B) TEM image of anti21-loaded DNA nanoclews (negatively stained with phosphotungstic acid). C) Size of DNA nanoclews obtained through RCA with various reaction time and the resultant FNCs. D) Entrapment efficiency of DNA nanoclews in (A).

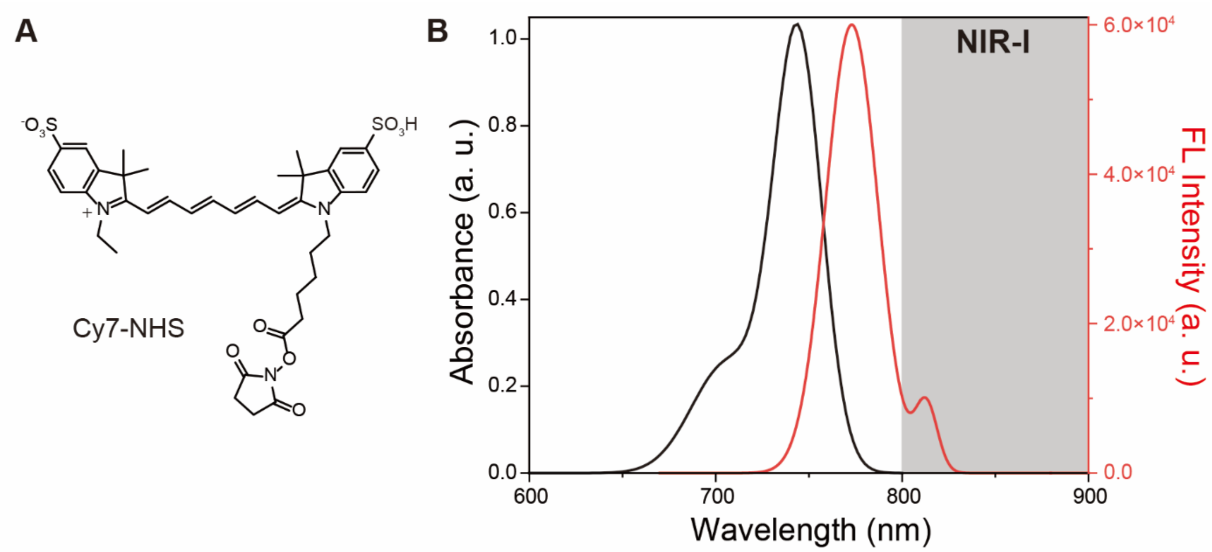

Figure S2. A) Chemical Structure of Cy7-NHS. B) Absorption and fluorescence emission spectra of anti21-Cy7 (40 μg mL^{-1}) in the far red and NIR-I region. The emission spectrum was collected under the excitation of 660 nm.

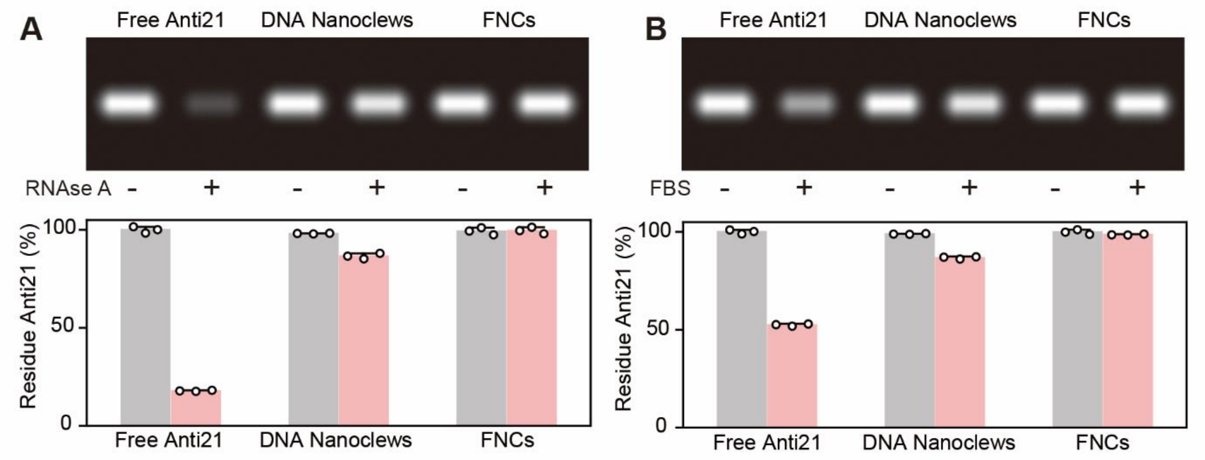

Figure S3. Stability of free anti21, anti21-loaded in DNA nanoclews or FNCs in the presence of RNAse A (A) and serum (FBS, B). Up: results of agarose gel retardation assay. Down: quantitative analysis. It should be noted that DNA fragments in the nanoclews would cover anti21 bands if the gel was stained with ethidium bromide and imaged in the visible region (common practice). Owing to the NIR-IIa emission of anti21-Cy7, we directly image the gel under 660 nm laser excitation and analyze the residue anti21 quantitatively.

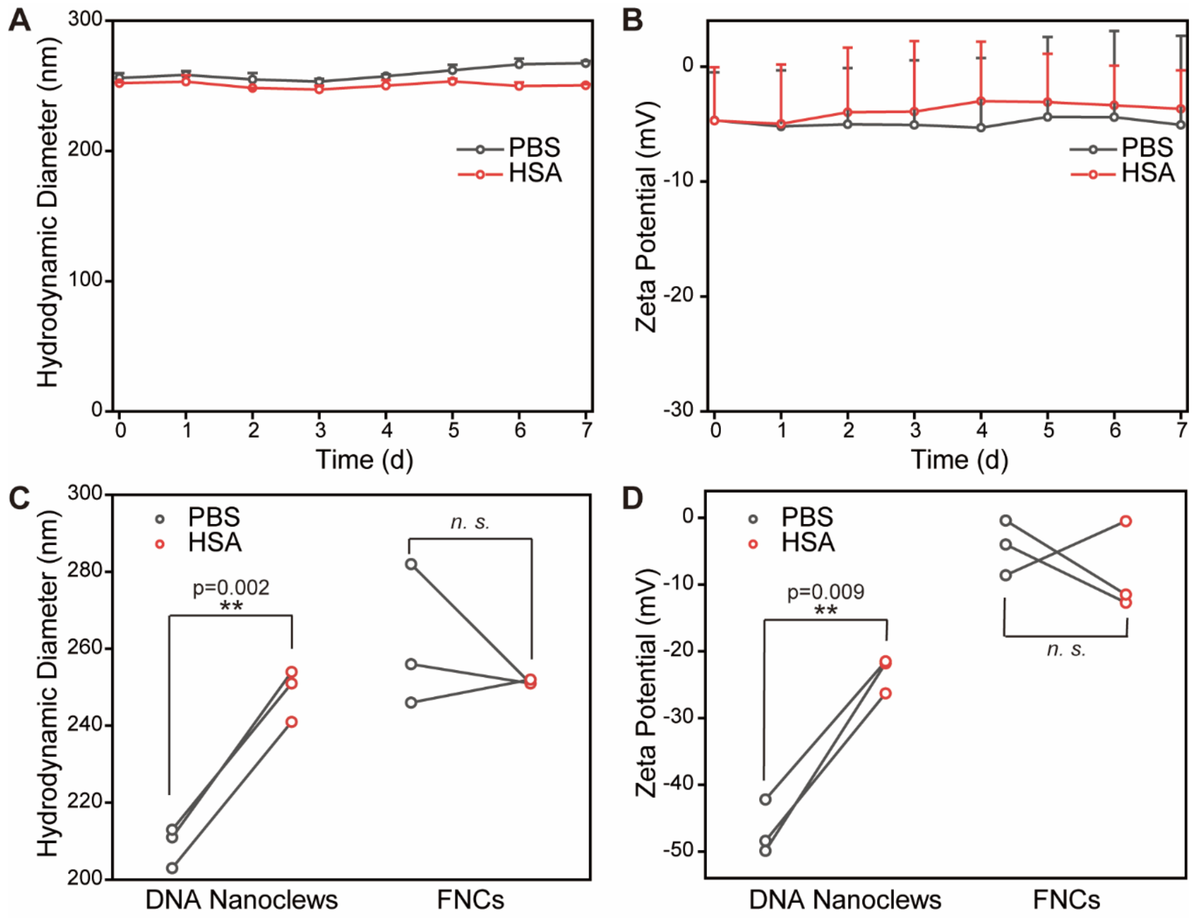

Figure S4. Hydrodynamic diameters (A) and zeta potentials (B) of FNCs at 25 °C for a week in HSA solution and PBS at pH 7.4. Hydrodynamic diameters (C) and zeta potentials (D) of DNA nanoclews and FNCs at 25 °C in PBS (pH 7.4) and 5 min after mixed with HSA solution.

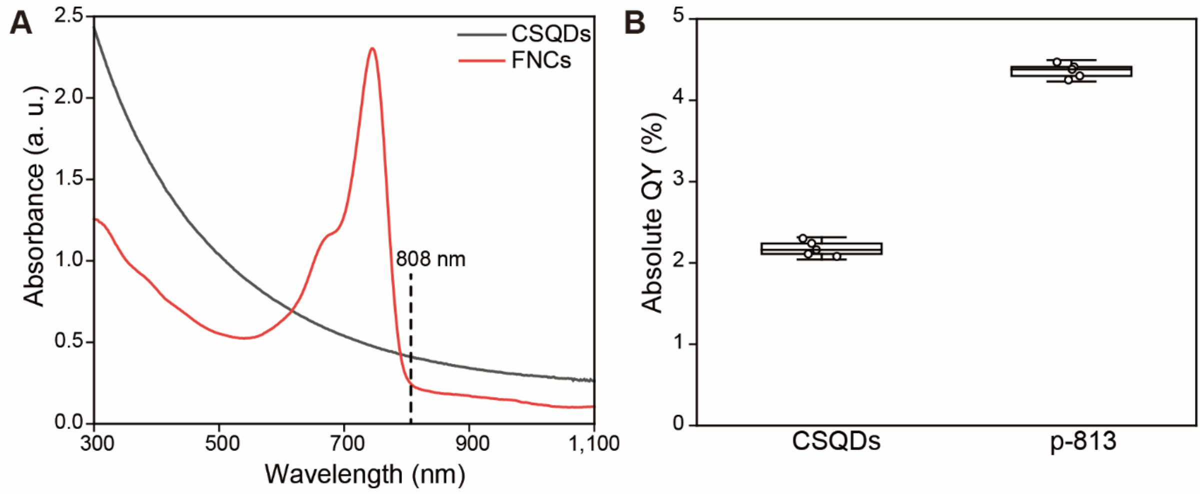

Figure S5. A) UV-vis spectra of CSQDs and FNCs in PBS (2 mg mL^{-1}). B) Absolute quantum yield of CSQDs and p-813 (emission range: 1000-1700 nm). Data are presented as box plots (center line, median; box limits, upper and lower quartiles; whiskers, SD; points, data).

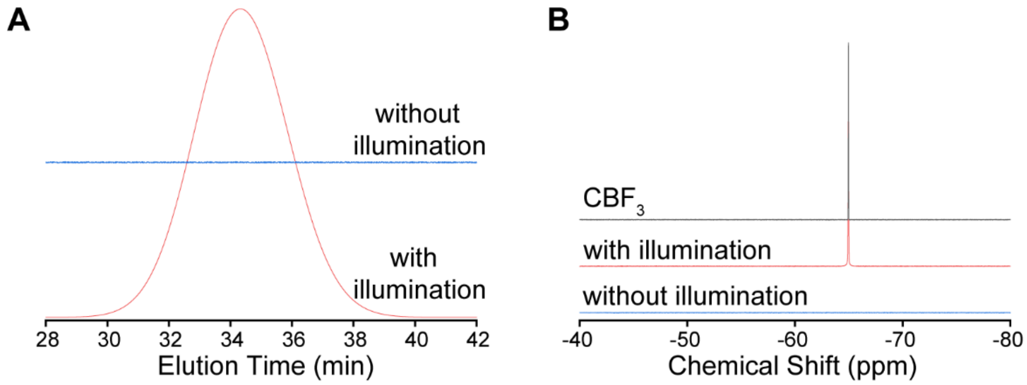

Figure S6. To verify fluoropolymer exfoliation, aqueous dispersion of FNCs is irradiated and ultrafiltrated with a 100 kDa centrifugal filter to remove the nanoclew core, CSQDs and large debris of $PCBF_3$ coatings. GPC trace of the filtrate (A) clearly indicates polymer debris after laser illumination, and fluorine peak with the same chemical shift as CBF_3 monomer (B) can be observed in the ^{19}F-NMR spectra. FNCs without irradiation are used as a negative control, and no polymer or fluorine content can be detected in the filtrate.

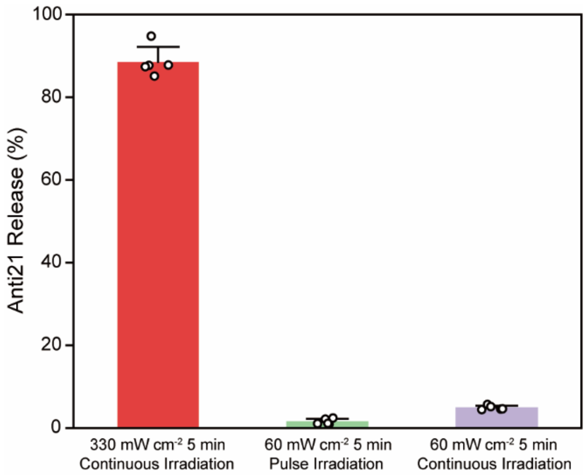

Figure S7. Anti21 release profiles of FNCs with NIR laser irradiation at various power density. Pulse irradiation is conducted by illuminating the sample for 100 ms and the pulse is given at a frequency of 1 Hz.

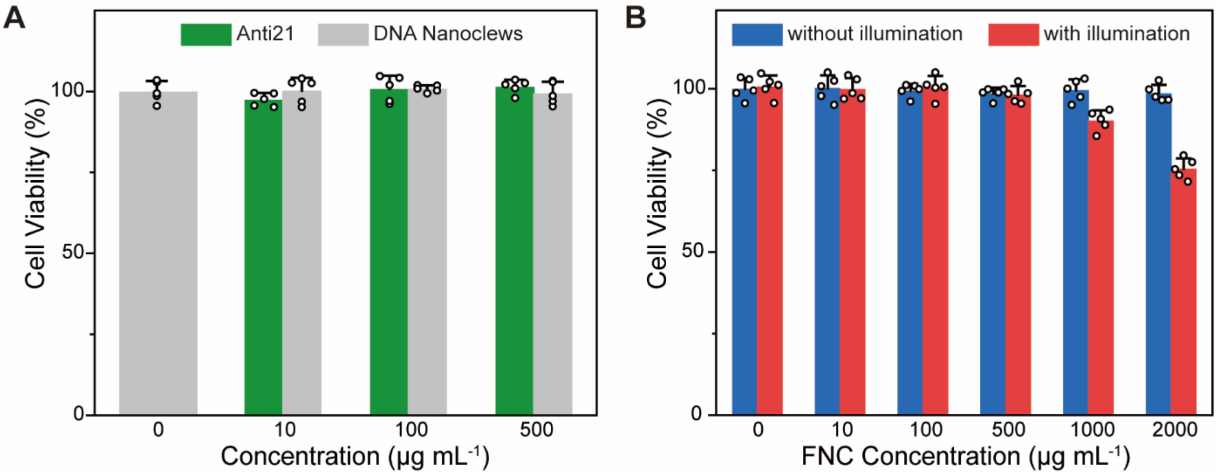

Figure S8. A) Cytotoxicity of anti21 and DNA nanoclews at various concentrations. B) Cytotoxicity and phototoxicity of FNCs at various concentrations.

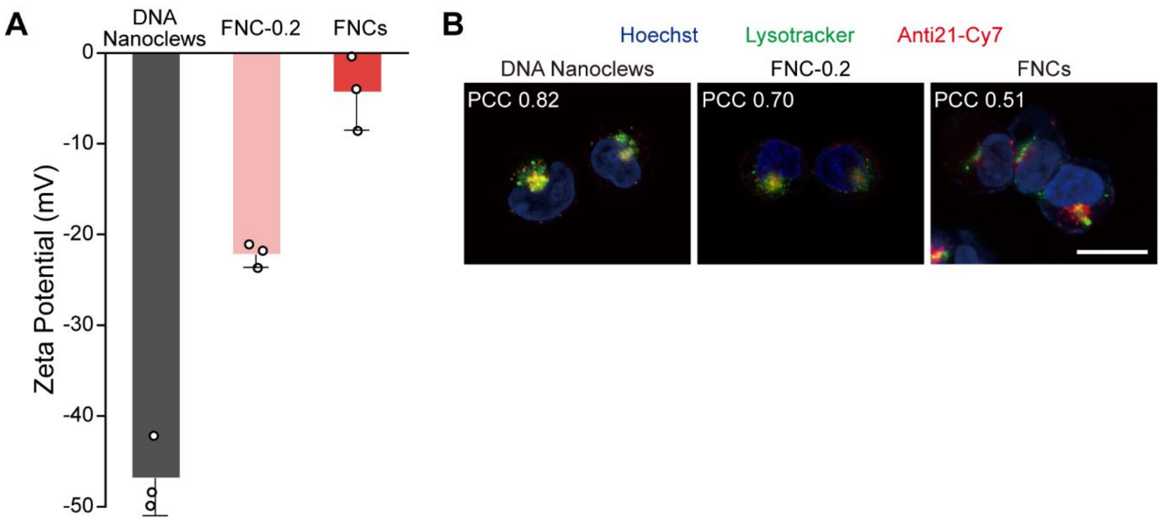

Figure S9. A) Zeta potentials of DNA nanoclews, FNC-0.2 (prepared by reducing CBF_3 used in polymerization to 1/5 of the original amount) and conventional FNCs. Zeta potential of FNC-0.2 is far lower than neutral, suggesting uncomplete covering of the zwitterionic polymer, and thus FNC-0.2 is less hydrophobic than conventional FNCs. B) Confocal microscopic images of MCF-7 cells incubated with DNA nanoclews, FNC-0.2 and FNCs. White bar indicates 20 μm in the image. Cells are costained with Lysotracker to observe the endosomal escape of cargo oligonucleotides. PCC is used to quantify the fluorescence signal overlap of Lysotracker and Anti21.

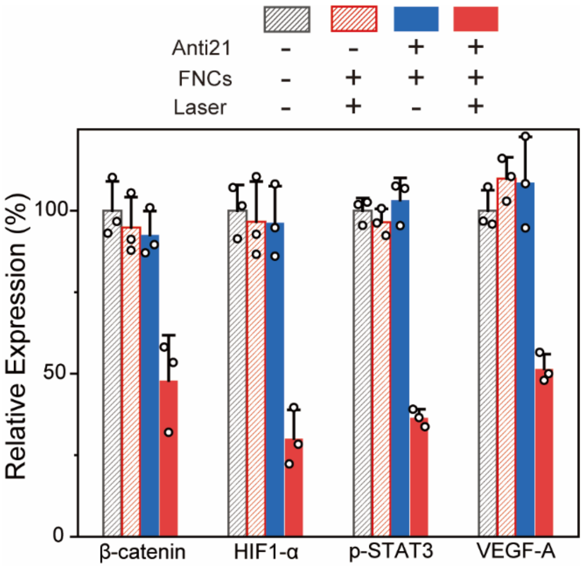

Figure S10. Quantification of protein expression level is shown by normalized gray values of western blot analysis in Figure 2F.

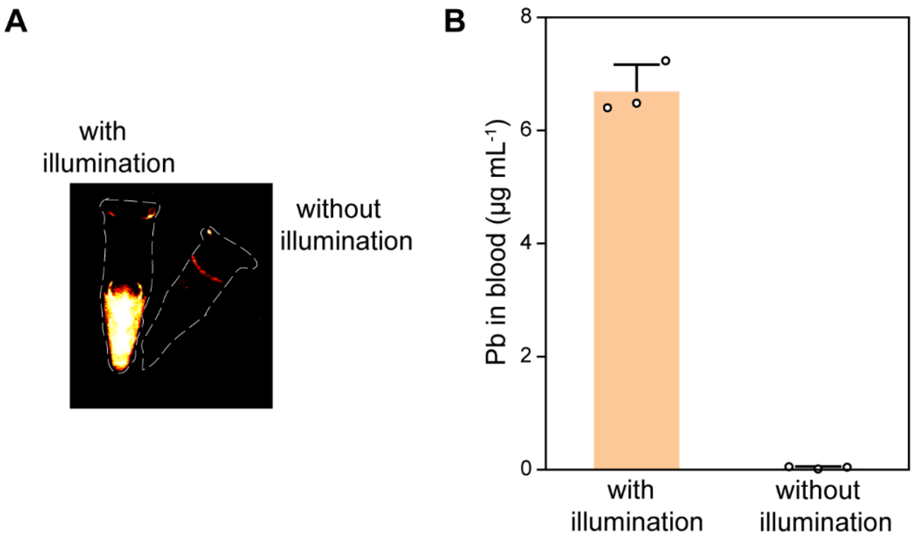

Figure S11 Analysis of blood samples collected from mice 8 hours after FNC administration. FNCs and blood cells are removed by centrifugation. No fluorescence (A) or Pb (B) can be detected in the supernatant, indicating no CSQD fall off from FNCs during circulation. Laser illumination of the blood samples induces FNCs deconstruction. In this case, CSQDs are released and can be detected in the supernatant by fluorescence imaging and ICP-OES.

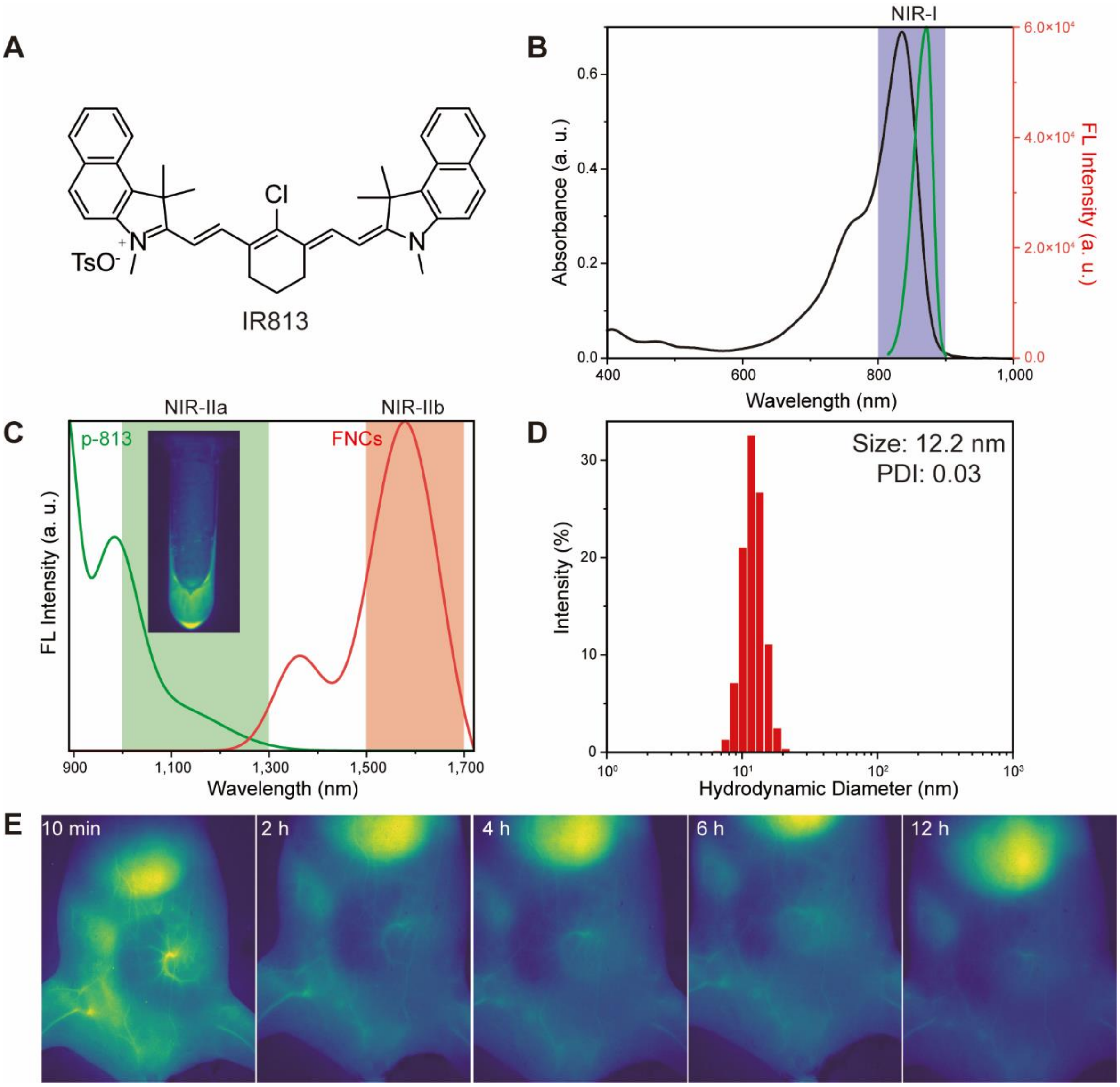

Figure S12. A) Chemical stucture of IR813 tosylate. B) Absorption and emission spectra of p-813 in the visble and NIR-I region. The fluorescence emission spectrum was collected under the exciation of 808 nm. C) NIR-II fluorescence spectra of p-813 and FNCs under the excitation of 808 nm laser. Inset: fluorescence image of a p-813 suspension in PBS collected in the NIR-IIa (1000-1300 nm) channel. D) Dynamic light scattering results of p-813. E) NIR-IIa imaging of nude mouse after intravenously injected with p-813. Vasculatures can be clearly observed within 12 h after administration so that the the circulating p-813 can be used to label vessels.

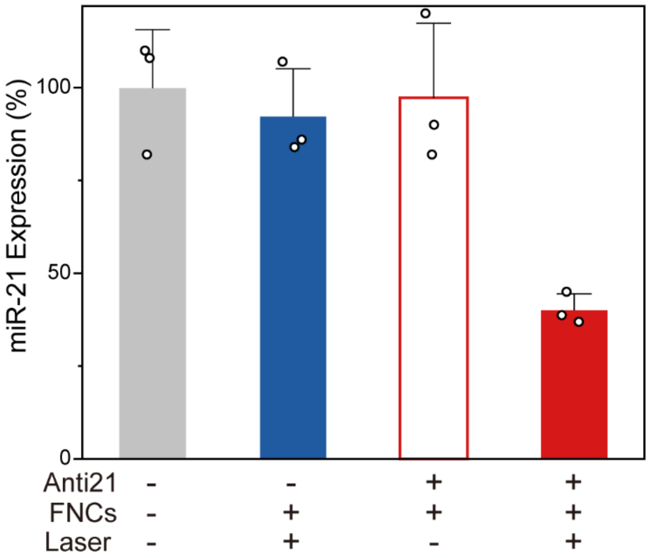

Figure S13. Relative miR-21 expression in tumor tissues after various treatments.

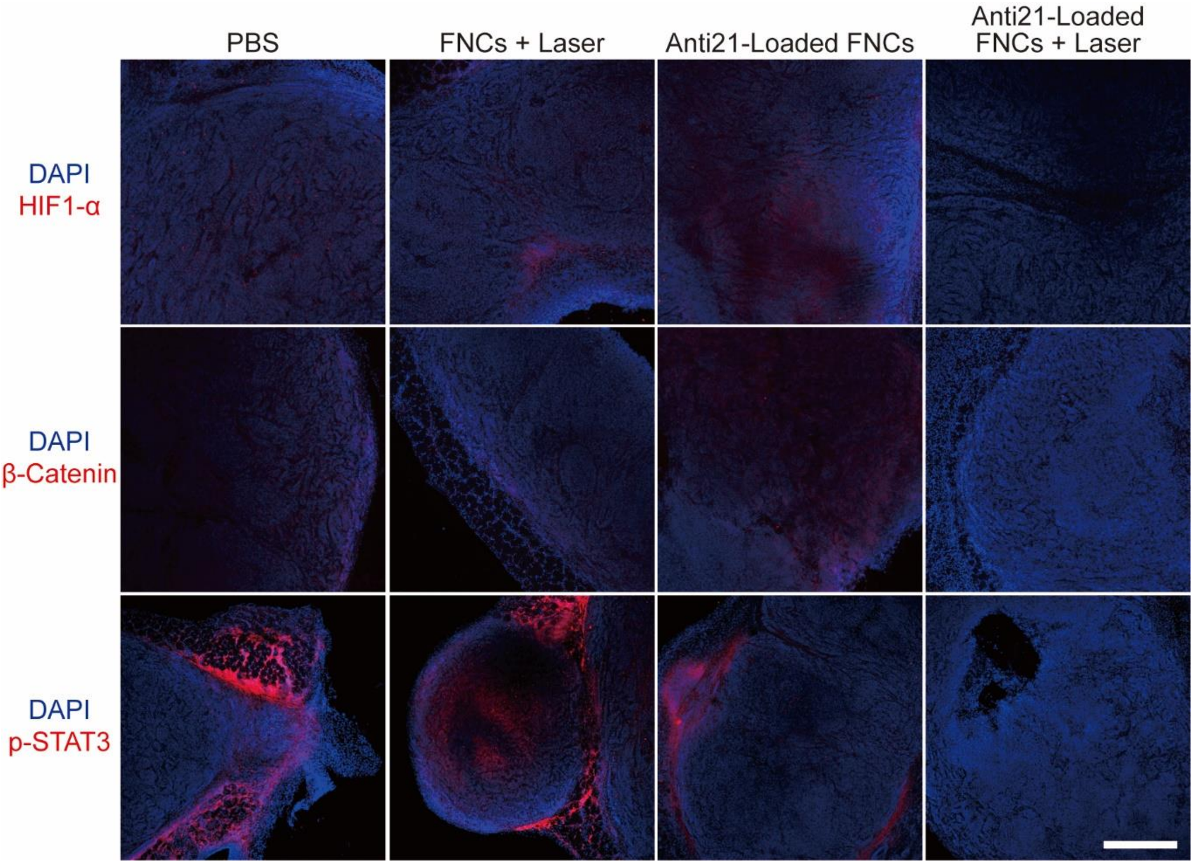

Figure S14. Immunostaining of HIF1-α, β-catenin and p-STAT3 in the tumor tissue. Scale bar: 0.5 mm.

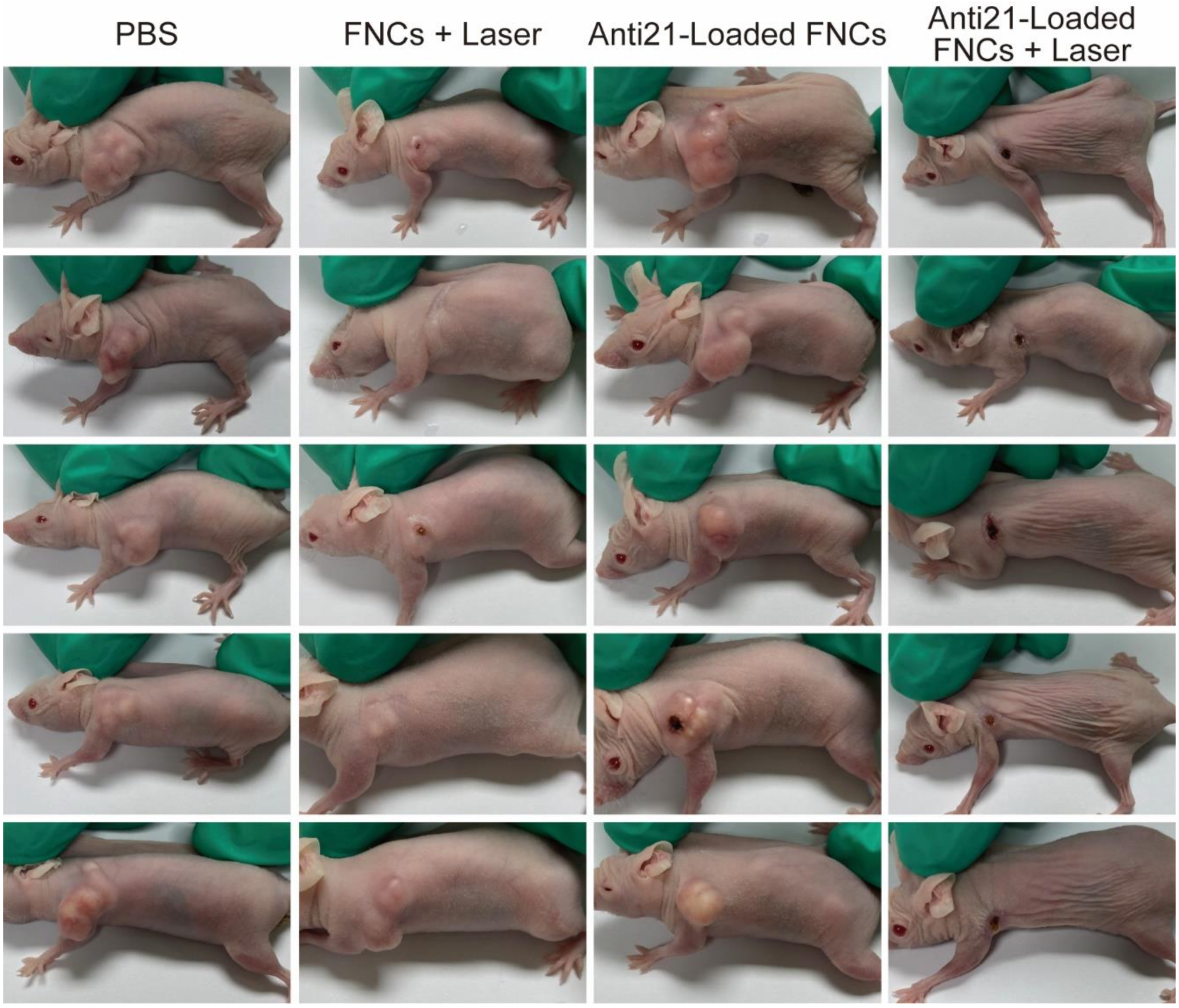

Figure S15. Images of mice 14 d after treatments.

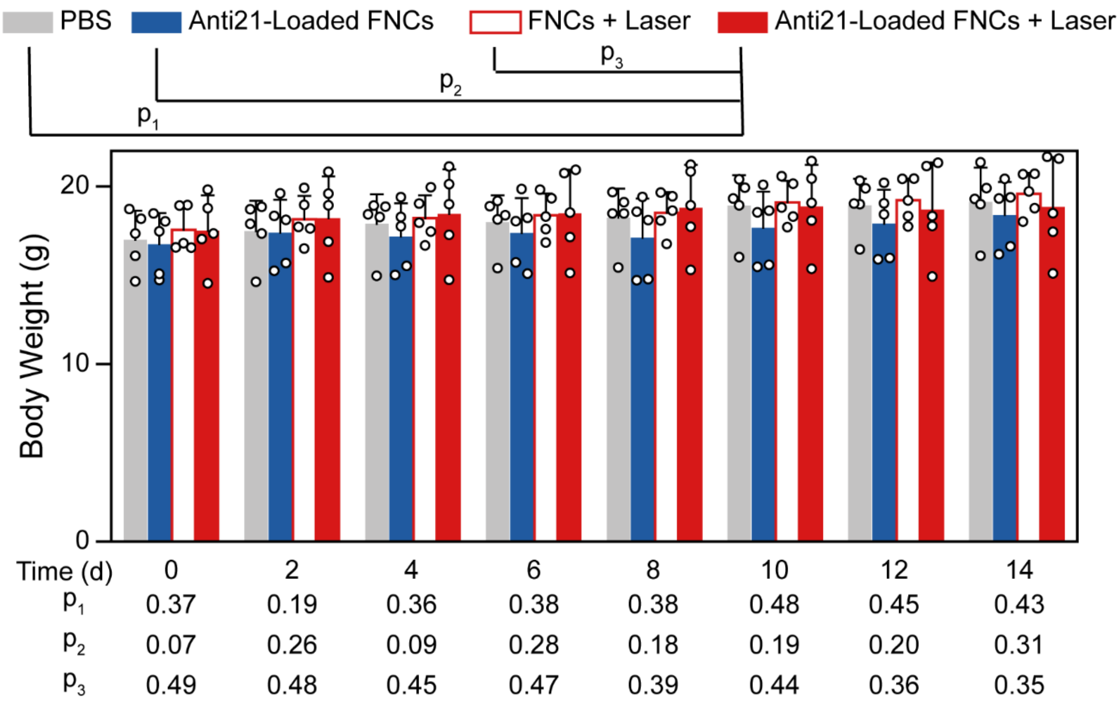

Figure S16. Body weight change of the mice and p values between groups.

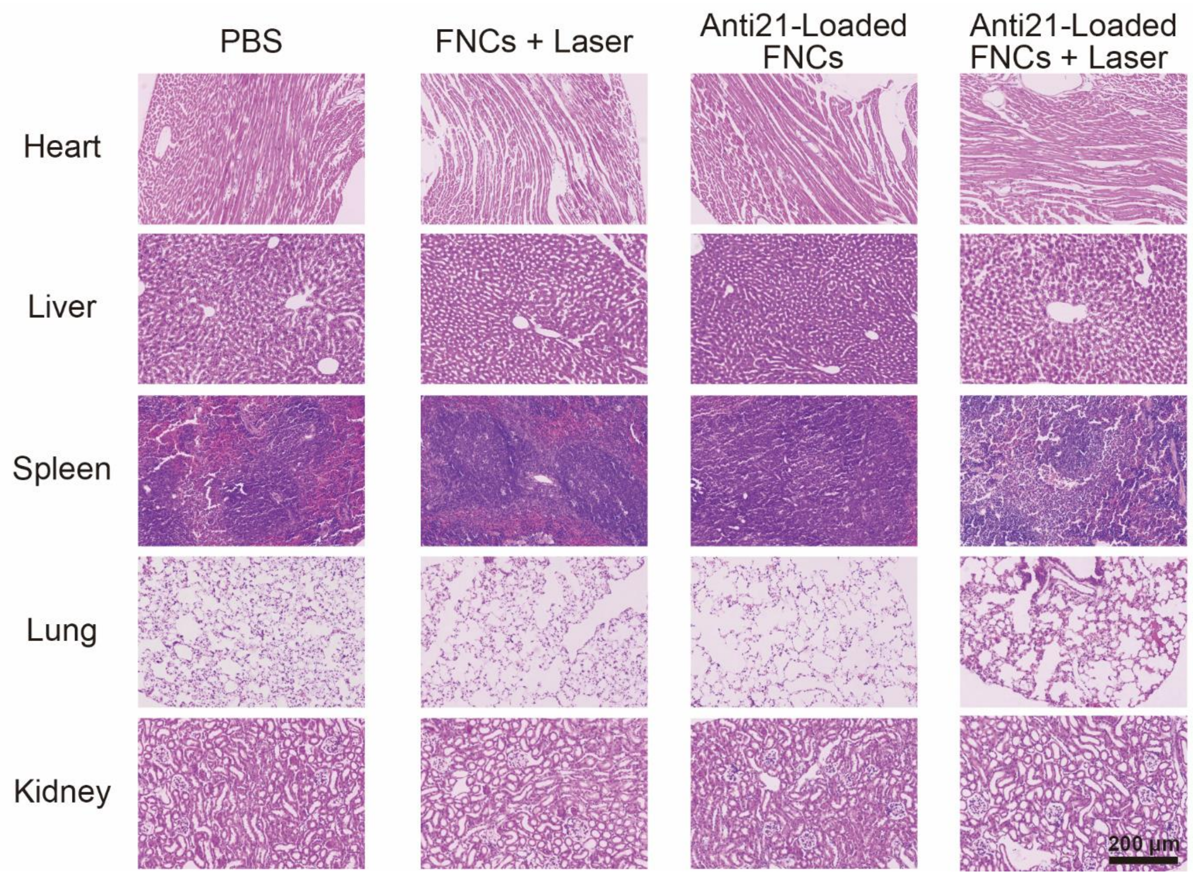

Figure S17. HE stained images of major organs collected from the mice.

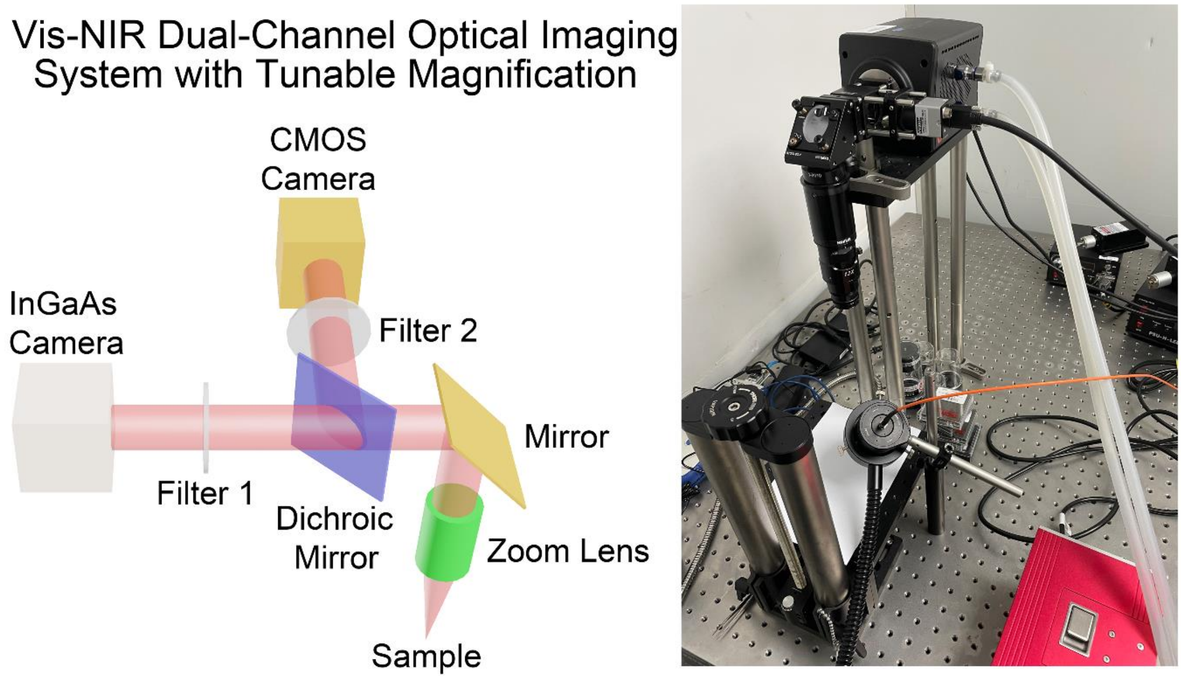

Figure S18. Schematic (left) and photograph (right) of the wide field NIR imaging setup used in this study.

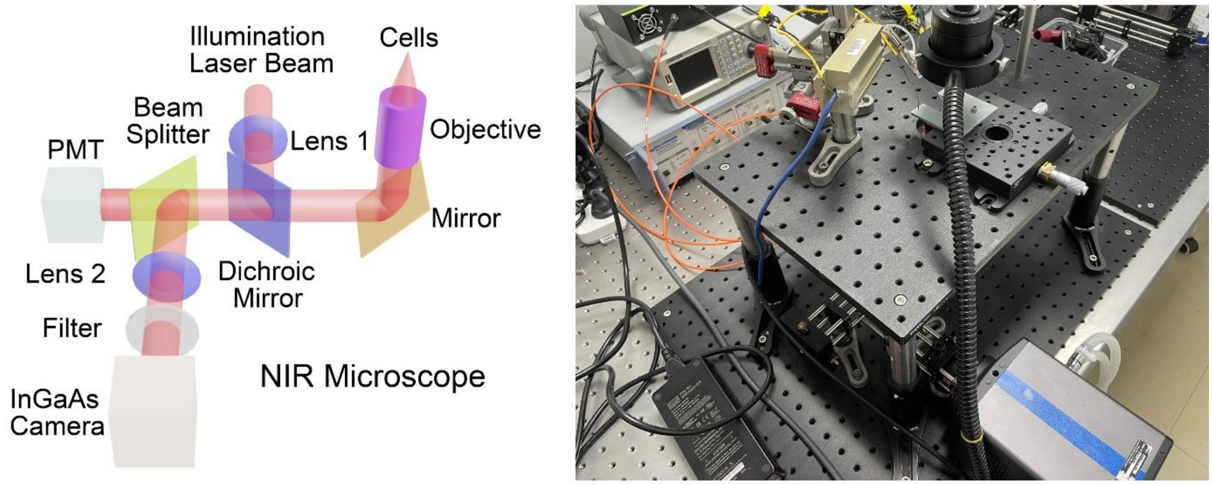

Figure S19. Schematic (left) and photograph (right) of the NIR microscope used in this study.

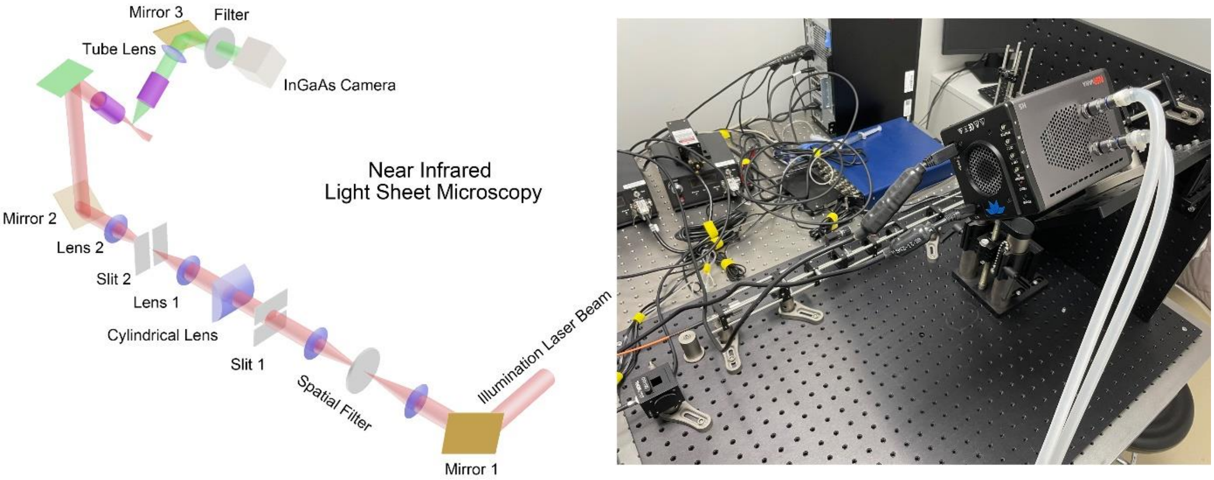

Figure S20. Schematic (left) and photograph (right) of NIR-LSM used in this study.

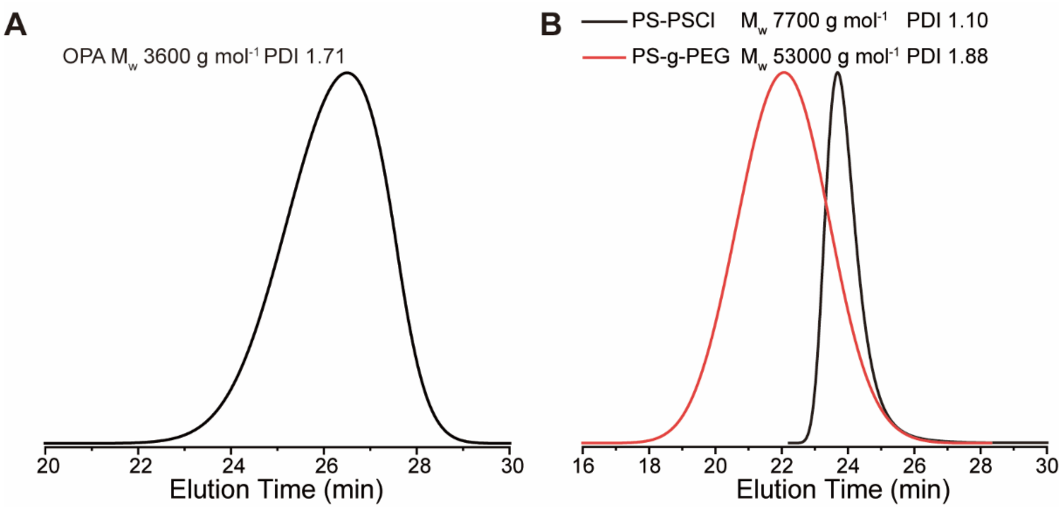

Figure S21. GPC characterization of OPA (A) and PS-g-PEG (B).

Table S1 Sequences of DNA oligos and miRNAs used in the study.

	Sequences (5'-3')
Template (**anti21 binding,** *panlindromic sequence*, ***primer binding***)	GCTAC**TCAACATCAGTC**ATCAATAA*GCTAGA TGCATCTAGC*AA***GCGCCGCCAC***TGATTTCACC GCTTCAA*GCTAGATGCATCTAGC*AAT
Primer	GTGGCGGCGC
Anti21	UCAACAUCAGUCUGAUAAGCUA-NH_2
Anti21 negative control	CAGUACUUUUGUGUAGUACAA-NH_2

Reference

[1] P. Kadhirvel, M. Azenha, E. Schillinger, M. R. Halhalli, A. F. Silva, B. Sellergren, *J. Chromatogr. A* **2014**, *1358*, 93.

[2] Z. Feng, Q. Li, W. Wang, Q. Ni, Y. Wang, H. Song, C. Zhang, D. Kong, X.-J. Liang, P. Huang, *Biomaterials* **2020**, *256*, 120184.

[3] M. Zhang, J. Yue, R. Cui, Z. Ma, H. Wan, F. Wang, S. Zhu, Y. Zhou, Y. Kuang, Y. Zhong, D.-W. Pang, H. Dai, *Proc. Nat. Acad. Sci. U.S.A.* **2018**, *115*, 6590.

[4] Y. Sun, Y. Jiao, Y. Wang, D. Lu, W. Yang, *Int. J. Pharm.* **2014**, *465*, 112.

[5] L. Cao, W. Yang, C. Wang, S. Fu, *J. Macromol. Sci., Part A: Pure Appl. Chem.* **2007**, *44*, 417.

[6] H. Wan, J. Yue, S. Zhu, T. Uno, X. Zhang, Q. Yang, K. Yu, G. Hong, J. Wang, L. Li, Z. Ma, H. Gao, Y. Zhong, J. Su, A. L. Antaris, Y. Xia, J. Luo, Y. Liang, H. Dai, *Nat. Commun.* **2018**, *9*, 1171.
